# Integrative eQTL-weighted hierarchical Cox models for SNP-set based time-to-event association studies

**DOI:** 10.1186/s12967-021-03090-z

**Published:** 2021-10-09

**Authors:** Haojie Lu, Yongyue Wei, Zhou Jiang, Jinhui Zhang, Ting Wang, Shuiping Huang, Ping Zeng

**Affiliations:** 1 Department of Biostatistics, School of Public Health, Xuzhou Medical University, Xuzhou, 221004 Jiangsu China; 2 Department of Biostatistics, School of Public Health, Nanjing Medical University, Nanjing, 211166 Jiangsu China; 3 Center for Medical Statistics and Data Analysis, Xuzhou Medical University, Xuzhou, 221004 Jiangsu China; 4 Key Laboratory of Human Genetics and Environmental Medicine, Xuzhou Medical University, Xuzhou, 221004 Jiangsu China

**Keywords:** Integrative analysis, SNP-set association study, Joint effect test, Hierarchical modeling, Cox model, Expression quantitative trait loci, Aggregated Cauchy association test

## Abstract

**Background:**

Integrating functional annotations into SNP-set association studies has been proven a powerful analysis strategy. Statistical methods for such integration have been developed for continuous and binary phenotypes; however, the SNP-set integrative approaches for time-to-event or survival outcomes are lacking.

**Methods:**

We here propose IEHC, an integrative eQTL (expression quantitative trait loci) hierarchical Cox regression, for SNP-set based survival association analysis by modeling effect sizes of genetic variants as a function of eQTL via a hierarchical manner. Three p-values combination tests are developed to examine the joint effects of eQTL and genetic variants after a novel decorrelated modification of statistics for the two components. An omnibus test (IEHC-ACAT) is further adapted to aggregate the strengths of all available tests.

**Results:**

Simulations demonstrated that the IEHC joint tests were more powerful if both eQTL and genetic variants contributed to association signal, while IEHC-ACAT was robust and often outperformed other approaches across various simulation scenarios. When applying IEHC to ten TCGA cancers by incorporating eQTL from relevant tissues of GTEx, we revealed that substantial correlations existed between the two types of effect sizes of genetic variants from TCGA and GTEx, and identified 21 (9 unique) cancer-associated genes which would otherwise be missed by approaches not incorporating eQTL.

**Conclusion:**

IEHC represents a flexible, robust, and powerful approach to integrate functional omics information to enhance the power of identifying association signals for the survival risk of complex human cancers.

**Supplementary Information:**

The online version contains supplementary material available at 10.1186/s12967-021-03090-z.

## Background

A wide range of recent genome-wide association studies (GWASs) have revealed that germline variants (i.e., single nucleotide polymorphisms [SNPs]) are also an important inherited component of cancer risk [1–3], although somatic mutations (e.g., copy number and DNA methylation alterations) play an essential role in the pathophysiology of many human cancers [4–6]. Conventionally, the association of SNPs is examined one at a time in cancer GWASs [1, 2]; however, the power for detecting such single SNP association signal remains limited because genetic variants generally have weak effect sizes [7–9], making the detection of cancer-associated SNPs difficult even with large samples. In addition, these identified genetic variants often explain only a very small fraction of cancer predisposition, leading to the so-called missing heritability [10–14], which also implies that a large amount of causal loci have yet been discovered and the endeavor to identify causative genes for cancers should continue.

As an effective alternative strategy, SNP-set analysis has been proposed in GWAS [15–21], where a set of SNPs defined a priori within a gene or other genetic units (e.g., pathway) are analyzed collectively to assess their joint influence on diseases or traits. Existing SNP-set approaches can be roughly grouped into two categories: (i) the burden test, in which the association with disease risk is evaluated for the overall effect of a weighted summation of variant alleles [22, 23]; and (ii) the variance component test, in which the association is examined for the variance of genetic variants under the framework of mixed-effects models [16, 24]. Due to the aggregation of multiple weak association signals and the reduced burden of multiple testing, SNP-set analysis is often more powerful than its counterpart of single SNP analysis. However, these SNP-set association approaches might be still underpowered when additional informative knowledge is available about the alternative. For example, if the association between a set of genetic variants and the survival risk of cancers is regulated through gene expression, the power improvement would be further achieved by integrating transcriptomic data into the test method. As it is widely demonstrated that disease-associated SNPs are more likely to be expression quantitative trait loci (eQTL) [25], it is thus conceivable that incorporating such knowledge would increase power for detecting association [26–28].

Several methods have been proposed for this goal within the mixed-effects model framework. For example, MiST was developed for continuous and binary phenotypes in rare variant association studies by modeling the effects of rare variants as a function of functional features while allowing for the heterogeneity of variant-specific effects [29]. This method was recently further generalized to integrate eQTL or other functional annotations [26, 30, 31]. Both simulations and real applications have exhibited the advantage of these integrative approaches compared with the general methods that do not incorporate functional characteristics of genetic variants. However, to our best knowledge, there is little relevant work with regards to integrative approaches for time-to-event association studies.

In the present study, we develop such a method within the hierarchical Cox model framework to jointly analyze multiple SNPs for association with censored survival outcomes (i.e., time-to-event phenotypes) [32, 33]. Specifically, we first group SNPs into SNP-sets based on a biologically meaningful unit (i.e., genes), and then test for the overall joint effects of all SNPs within the gene. To integrate eQTL, following prior work [26, 29], we suppose the effect sizes of SNPs are partly explained by eQTL via a hierarchical modeling. As a result, our association analysis consists of two components: the first component stands for the fixed effect through the weighted burden score to reflect the impact of genetic variants on the survival risk explained by eQTL, while the second component examines the residual effects of genetic variants beyond eQTL. These residual effects are treated as random effects following an arbitrary distribution with mean zero and variance τ [32, 33]. Therefore, methodologically, testing the joint effect for a group of SNPs with the survival risk of a cancer of focus is equivalent to examining the fixed effect and random effects simultaneously.

Under our model context, a novel decorrelated modification is made so that two independent statistics (i.e., a burden test statistic and a variance component test statistic) are derived for each of the two components. Then, the joint effect test can be easily constructed based on these two uncorrected statistics via various p-values combination strategies. To this aim, we consider three data-driven approaches (e.g., the Fisher’s combination, the optimally weighted combination, and the adaptively weighted combination) for combining them to capture the association signals from both sources. To further enhance power, we exploit the recently developed aggregated Cauchy association test (ACAT) to integrate the strengths of all the five types of test methods (i.e., three combination tests as well as the burden test and the variance component test; the latter was also called the kernel machine [KM] test) [34, 35]. We refer to our proposed approach and test framework described above as integrative eQTL hierarchical Cox model (IEHC). Extensive simulations demonstrate that the three combination tests have comparable power or are better than both the burden and variance component tests under some specific scenarios, while IEHC-ACAT enjoys consistently higher power across all simulation scenarios. We finally apply IEHC to ten TCGA cancers which have one explicit relevant tissue in The Genotype-Tissue Expression (GTEx) project and integrate eQTL into our method [36]. We identified a total of 21 (9 unique) cancer-associated genes which would otherwise be missed by the general SNP-set based survival association methods that do not consider eQTL.

## Methods

### An overview of the IEHC model and the joint test

First, consider that there are *S* genotypes (denoted by G_*i*_ and coded as 0, 1 or 2 in terms of the number of effect allele) of SNPs located within a given gene and *p* covariates X_*i*_ (e.g., age, gender, and cancer stage) for *n* individuals; and *S* in general varies gene by gene. In addition, denote the observed survival time by *t*_*i*_ and the true survival time by *T*_*i*_ with *d*_*i*_ indicating the censored status; that is, *d*_*i*_ = 1 if *T*_*i*_ = *t*_*i*_, whereas *d*_*i*_ = 0 if *T*_*i*_ < *t*_*i*_. Under the proportional hazards condition, we assume the hazard function λ(*t*) of the survival time *t*_*i*_ is related to G_*i*_ and X_*i*_ through the classical Cox model [37]

$$\log \left( {\frac{{{\uplambda }(t_{i} )}}{{{\uplambda }_{0} (t_{i} )}}} \right)\; = \;{\text{G}}_{i}^{T} {\varvec{\alpha}}\;{ + }\;{\text{X}}_{i}^{T} {\varvec{c}}$$

where λ_0_ is an arbitrary baseline hazard function, ***α*** = (*α*_1_, …, *α*_*S*_) is an *S*-vector of effect sizes for SNPs and ***c*** = (*c*_1_, *c*_2_, …, *c*_*p*_) is a *p*-vector of fixed effect sizes for clinical covariates.

We here only provide an overview of IEHC, with technical details demonstrated in the Additional file 1. In brief, IEHC examines a group of SNPs in one gene at each time and integrates eQTL information by extending the Cox model above in a hierarchical manner

$$\begin{aligned} \log \left( {\frac{{{\uplambda }(t_{i} )}}{{{\uplambda }_{0} (t_{i} )}}} \right)\; & = \;\sum\limits_{j\; = \;1}^{S} {{\text{G}}_{ij} \alpha_{j} } \;{ + }\;\sum\limits_{k\; = \;1}^{p} {X_{ik}^{T} c_{k} } \; = \;\eta \;{ = }\;{\text{G}}_{i}^{T} {\varvec{\alpha}}\;{ + }\;{\text{X}}_{i}^{T} {\varvec{c}} \\ \alpha_{j} \; & = \;\beta_{j} \; \times \;\theta \; + \;b_{j} \\ b_{j} \; & \sim \;N(0,\;{\uptau }) \\ \end{aligned}$$

Of note, plugging *α*_*j*_ into the first line leads to $\eta \;{ = }\;({\text{G}}_{i}^{T} {\varvec{\beta}})\theta \;{ + }\;{\text{G}}_{i}^{T} {\varvec{b}}\;{ + }\;{\text{X}}_{i}^{T} {\varvec{c}}$. In the above, *β*_*j*_ is the known eQTL effect size of the *j*^th^ SNP and directly obtained in terms of summary statistics from the GTEx project [36, 38], *θ* is a scale of coefficient for eQTL and quantifies the association between the survival risk and the weighted burden score ${\text{G}}_{i}^{T} {\varvec{\beta}}$, and *b*_*j*_ is the normal residual variant-specific effect size that is not interpreted by eQTL alone. Then, the hypothesis of no association between a set of SNPs and the survival outcome is

$$H_{0} :\;\theta \; = \;0\;{\text{and}}\;b\; = \;0\; \Leftrightarrow \;H_{0} :\;\theta \; = \;0\;{\text{and}}\;{\uptau }\; = \;0$$

This is a joint test including both fixed effect and random effects: the first component examines the influence of genetic variants on the survival risk explained by eQTL (i.e., *θ* = 0); while the second component examines the impact of genetic variants beyond the effects of eQTL (i.e., τ = 0).

To implement the hypothesis testing while circumventing the potential correlation between statistics and improving the statistical computation, we propose the following two-stage strategy. Briefly, we derive the test statistic for *θ* under *H*_0_: *θ* = 0 and τ = 0 as usual, while derive the score statistic for τ under τ = 0 but without the constraint of *θ* = 0. By doing this, we ensure that these two statistics are independent (see simulation results in Additional file 1). This strategy substantially eases the construction of test statistics for the joint test and two asymptotically independent statistics are eventually derived: one for *θ* in the general Cox model (say *U*_*θ*_) [37] and the other for the variance component parameter τ in the kernel machine (KM) Cox model (say *U*_*τ*_) [32, 33]. We combine the two uncorrelated statistics via several aggregation approaches, including the Fisher’s combination (IEHC-Fisher) [39, 40], the optimally weighted linear combination (IEHC-optim) as well as the adaptively weighted linear combination (IEHC-adapt). For IEHC-optim we establish *T*_*ρ*_ = *ρU*_*θ*_ + (1-*ρ*)*U*_*τ*_, with $\rho \; \in \;[0,\;1]$ controlling the contribution of the fixed-effect component. The final *ρ* in IEHC-optim is selected by optimizing *T*_*ρ*_. On the other hand, IEHC-adapt is a data-adaptive generalization of the Fisher’s combination [39, 40], for which the test statistic takes the form *T* = *ρ*_*θ*_*Z*_*θ*_ + *ρ*_*τ*_*Z*_*τ*_, where *Z*_*θ*_ = − 2log(*p*_*θ*_) and *Z*_*τ*_ = − 2log(*p*_*τ*_), based on which *ρ*_*θ*_ and *ρ*_*τ*_ are determined via an adaptive manner.

The IEHC test described above includes two special cases: the burden test for examining the fixed effect *θ* (with τ = 0) in the general Cox model and the KM test examining the variance component parameter τ (with *θ* = 0) in the KM Cox model. To further boost the power, we employ the recently developed aggregated Cauchy association test (ACAT) to combine the strengths of these five methods (i.e., the burden test, the KM test and three joint tests including IEHC-Fisher, IEHC-optim and IEHC-adapt) [34, 35]. The advantage of IEHC-ACAT is that it allows us to aggregate correlated p-values obtained from multiple various tests into a single well-calibrated p-value while maintaining the type I error control correctly. The detailed procedures for these approaches are relegated to Additional file 1. The code for IEHC is freely available at https://github.com/biostatpzeng/IEHC.

### Simulations for type I error control and power evaluation

We now perform simulations to evaluate the type I error control and power for IEHC. To mimic the truth, we undertook simulations based on realistic genotypes available from the Geuvadis program because the sample size in the real-life applications used in this paper matched closely that of Geuvadis [41]. First, we obtained 550 a group of correlated SNPs in a local genetic region from 465 individuals in Geuvadis. During the simulation we randomly selected *S* nearby SNPs (denoted by ***G***_1_), with *S* varying according to a uniform distribution ranging from 20 to 50 (i.e., *S* was on average equal to 35); among these selected genetic variants we further randomly set 0%, 30% or 50% of SNPs having zero effect sizes. We generated the gene expression level with the first 165 individuals and sampled the effect sizes ***β*** from a normal distribution with a special variance so that the proportion of the explained variation (PVE) of the expression level would be 30% or 50%.

Then, we calculated ***α*** = ***β*** × *θ* + ***b***, with ***b*** following a normal distribution with variance τ. Two independent covariates (i.e., *X*_1_ was binary and *X*_2_ was continuous) were also generated with each having an effect size of 0.50. We employed the inverse probability method to generate the survival time which followed a Weibull distribution with the shape parameter equal to 1 and the scale parameter equal to 0.01 [42]. The location parameter (denoted by *μ*) of this Weibull distribution was determined by ***α*** and the two covariates: *μ* = exp(*η*) and *η* = ***G***_2_***α*** + 0.5*X*_1_ + 0.5*X*_2_, with ***G***_2_ representing the remaining genotypes of 300 samples in Geuvadis. The censored rate was fixed to be 50% in a random manner. Note that, this relatively high censored rate corresponded to the similar situation observed in the TCGA cancer dataset (see below). We set *θ* = 0 and τ = 0 to assess the type I error control and run 10^5^ replications. To evaluate the power, we specified *θ* = 0, 0.1, 0.2, 0.3 or 0.4, and τ = 0, 0.02, or 0.04 (here at least one of *θ* and τ was nonzero). The power simulation was repeated 10^3^ times.

### TCGA cancers and GTEx eQTL summary statistics

#### TCGA cancers and quality control

We applied the proposed method to multiple cancer data publicly available from TCGA [43]. We downloaded these datasets at https://xenabrowser.net/ and focused on cancers having one explicitly relevant tissue in the GTEx project [36]. However, we did not include PRAD (prostate adenocarcinoma) and THCA (thyroid carcinoma) as nearly all the PRAD patients (99.3% = 146/147) and THCA patients (95.7% = 315/329) were alive during the follow-up. We also removed DLBC (lymphoid neoplasm diffuse large b-cell lymphoma), KICH (kidney chromophobe) and TGCT (testicular germ cell tumor) because of too small sample sizes (i.e., only 24 for DLBC, 57 for KICH and 69 for TGCT). Finally, we reserved ten cancers for further analysis (Table 1).

**Table 1:** Summary information of 10 TCGA cancers and the number of genes, sample sizes and SNPs of these cancers after combining the tissue in GTEx and quality control

Cancer	*N*_0_	*N*_1_	*m*	Age	Female/male	Censored rate (%)	Stage or grade (1/2/3/4/5)	Tissue in GTEx	*N*_2_	*k*_0_	*k*_1_
ACC	97	75	4,473,001	47.6 ± 16.5	50/25	60.0	8/33/16/18/0	Adrenal gland	175	11,822	11,165
BRCA	1283	736	2,281,892	58.8 ± 13.0	736/0	85.9	138/408/174/11/5	Breast mammary tissue	251	13,068	7,793
COAD	570	201	4,216,239	66.0 ± 13.0	97/104	75.1	34/78/62/27/0	Colon transverse	246	12,779	11,586
LIHC	469	166	3,369,784	62.7 ± 14.1	73/93	60.8	79/44/39/4/0	Liver	153	11,073	8,595
LUAD	877	384	3,831,580	65.9 ± 9.9	213/171	63.0	216/90/62/16/0	Lung	383	13,300	10,617
LUSC	765	344	3,279,532	67.1 ± 8.8	94/250	57.3	176/117/48/3/0	Lung	383	13,300	9,450
OV	758	455	1,527,607	60.2 ± 11.4	455/0	37.4	9/19/353/74/0	Ovary	122	12,623	7,851
PAAD	223	159	4,679,901	65.6 ± 10.8	69/90	45.9	20/130/4/5/0	Pancreas	220	11,173	10,728
STAD	544	249	3,384,736	64.8 ± 10.2	97/152	60.2	33/75/128/13/0	Stomach	237	12,045	9,247
UCEC	605	368	4,008,620	64.4 ± 10.8	368/0	83.2	239/33/79/17/0	Uterus	101	12,592	10,948

*N*_0_: the initial sample size in TCGA; *N*_1_: the sample size after quality control; *m*: the number of SNPs after combination; *N*_2_: the sample size in GTEx; *k*_0_: the number of genes after combination; *k*_1_: the number of genes after quality control

To avoid the issue of ethnic heterogeneity, we included only patients of European ancestry and selected the overall survival time and status in our analysis following prior work [44]. Several important clinical covariates were incorporated, such as age, gender, and pathologic tumor stage because only these clinical variables were available for the majority of TCGA patients. When the pathologic tumor stage is unavailable, we alternatively employed the clinical stage (i.e., OV). We further standardized each clinical covariate. In addition, for every cancer we only kept samples from primary tumor tissues and excluded patients with too many missing values in clinical covariates (Table 1).

#### TCGA genotypes, imputation, and quality control

For each cancer we first filtered out SNPs that had missingness rate > 0.95 across patients, genotype calling rate < 0.95, minor allele frequency (MAF) > 0.01, or Hardy–Weinberg equilibrium (HWE) p-value < 10^–4^. Then, we undertook imputation by first phasing genotypes with SHAPEIT [45], then imputed SNPs based on the Haplotype Reference Consortium panel [46] on the Michigan Imputation Server using minimac3 [47]. The filtering procedure for imputed genotypes included HWE p-value < 10^–4^, genotype call rate < 95%, MAF < 0.01 and imputation score < 0.30.

#### GTEx eQTL summary statistics and the combination with TCGA

At the same time, for these kept cancers we obtained eQTL summary statistics of the related tissue from GTEx [36] and performed a stringent quality control (Table 1): (i) reserved SNPs with MAF > 0.05; (ii) excluded non-biallelic SNPs and SNPs with strand-ambiguous alleles; (iii) excluded SNPs that had no rs labels as well as duplicated ones; (iv) kept only SNPs which were included within TCGA; (v) removed SNPs whose alleles did not match those in TCGA; (vi) aligned the effect allele of SNP between TCGA and GTEx.

For comparison we implemented the following six methods in both simulations and real-life applications within the context of Cox modeling log[λ(*t*)/λ_0_(*t*)/] = *η*: (i) the burden test: to examine *H*_0_: *θ* = 0 in *η* = (**G*β***) × *θ* + **X*c*** using the Wald test in the general Cox model; (ii) the KM test: to assess *H*_0_: τ = 0 in *η* = **G*b*** + **X*c*** and *b* ~ *N*(0, τ) using the kernel-machine based approach; (iii) IEHC-Fisher: to jointly test *H*_0_: τ = 0 and *θ* = 0 in *η* = (**G*β***) × *θ* + **G*b*** + X*c* and *b* ~ *N*(0, τ) using the Fisher’s combination method, or (iv) IEHC-adapt using the adaptive combination method, or (v) IEHC-optim using the optimal combination method; (vi) IEHC-ACAT: to aggregate the first five tests using the Cauchy combination method.

## Results

### Independence of the two statistics in the joint test and type I error control

**Fig. 1:**
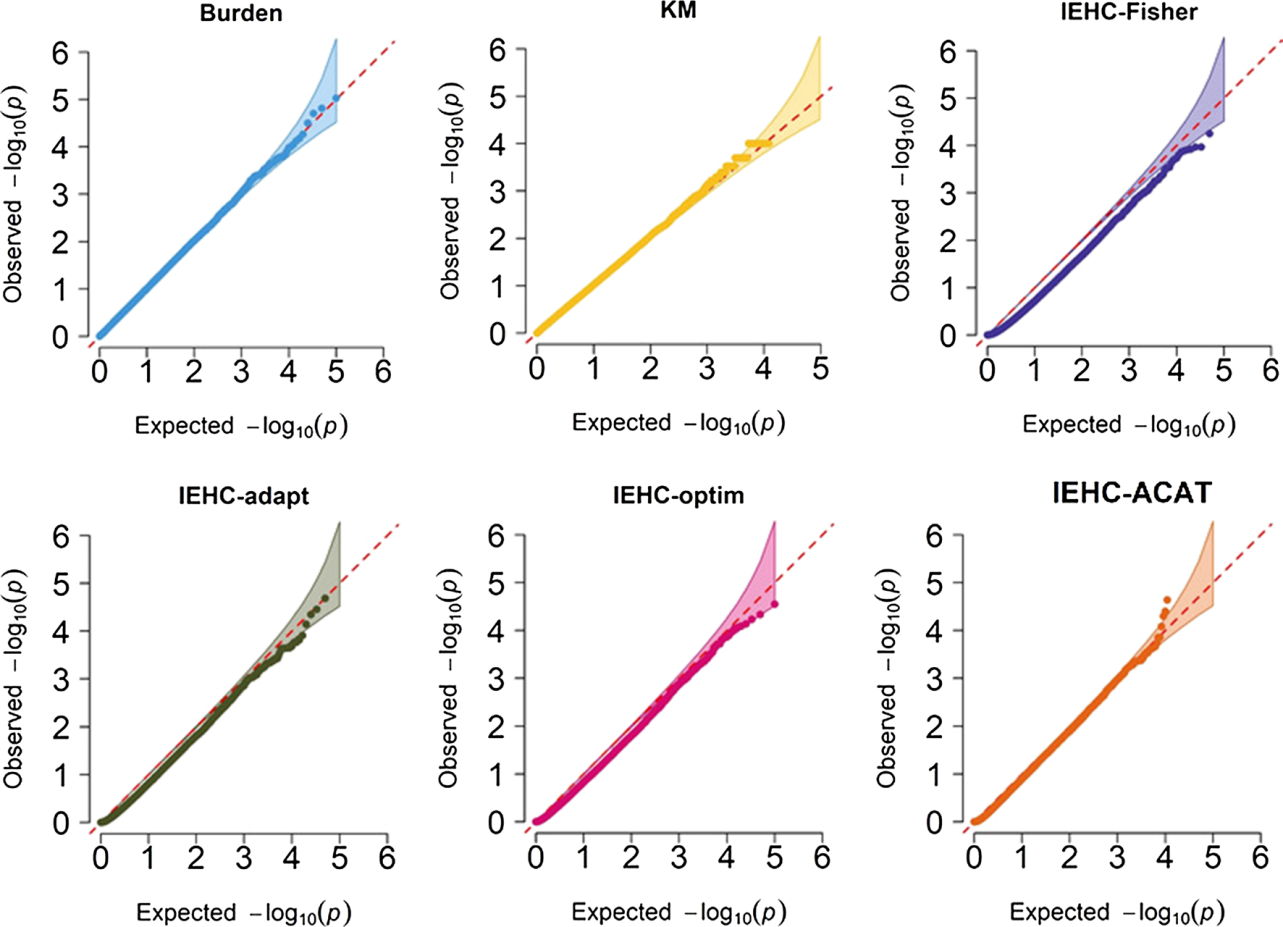
The QQ plots evaluating the type I error for the burden test, the KM test, IEHC-Fisher, IEHC-adapt, IEHC-optim as well as IEHC-ACAT under the null that both *θ* and τ are zero

First, in order to validate the independence of the two statistics (denoted by *U*_*θ*_ and *U*_*τ*_) constructed in the joint test of IEHC, we computed the Pearson’s correlation coefficient between them under the null of our simulation and find little evidence supporting the dependence of the two statistics (Additional file 1: Figure S1). For instance, across the 10^5^ replications, the overall correlation between *U*_*θ*_ and *U*_*τ*_ is 1.75 × 10^–3^ (95% confidence interval: – 4.44 × 10^–3^—7.95 × 10^–3^, *P* = 0.580), confirming the validity of our proposed joint test framework within which we can combine two uncorrelated statistics in a statistically straightforward fashion. Next, the Q-Q plots demonstrate all the tests, including the burden test, the KM test, IEHC-Fisher, IEHC-adapt, IEHC-optim as well as IEHC-ACAT, effectively control the type I error (Fig. 1). Particularly, we find IEHC-ACAT correctly maintains the type I error control even if the aggregated test methods (i.e., the first five) are highly correlated (Additional file 1: Figure S2). Furthermore, IEHC-Fisher is more powerful when the fixed effect explained by eQTL and random effects beyond eQTL exist simultaneously, but is less powerful when only one of the two types of effects is true or under the null that both *θ* and τ are zero, where the deflated p-values are observed (Fig. 1).

### Simulation results for power evaluation

**Fig. 2:**
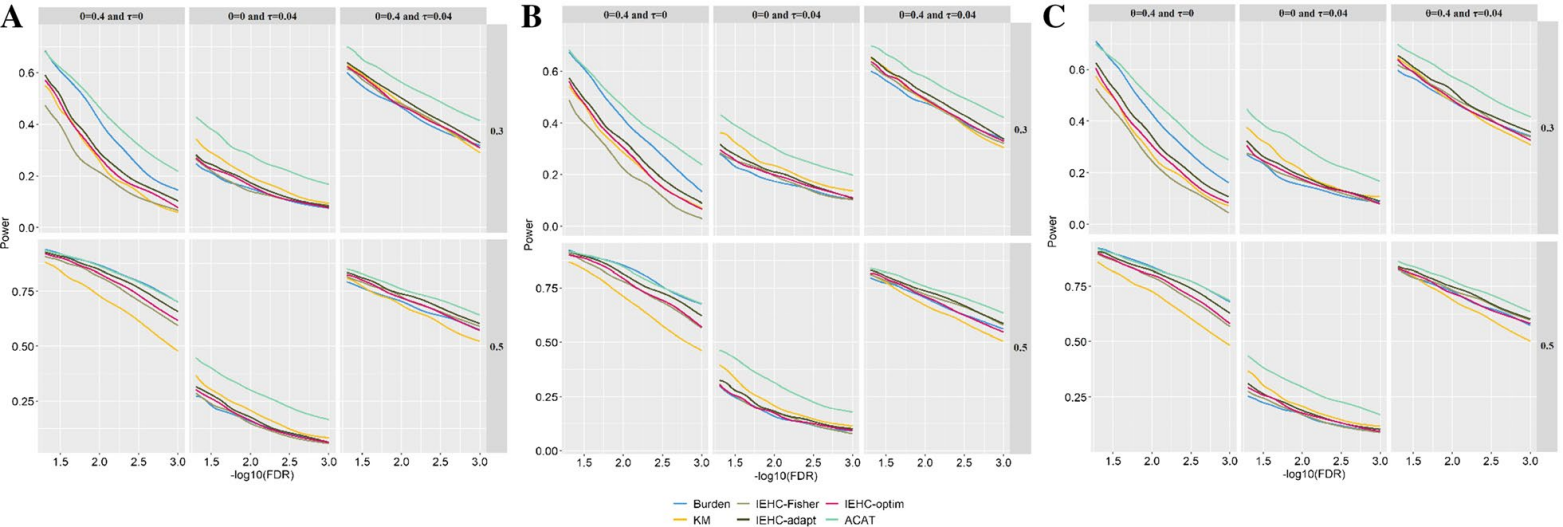
Power comparison among the six test methods under the alternative. In the simulation scenarios, 30%, 50% or 0% SNPs were randomly selected to have zero effect sizes. The PVE of the expression level explained by ***β*** was set to 0.3 (above) or 0.5 (below). **A** 30% SNPs having zero effect sizes; **B** 50% SNPs having zero effect sizes; **C** 0% SNPs having zero effect sizes. Here, *θ* = 0.4 or (and) τ = 0.04

We now compare the power of these tests under the alternative. To save the space, here we only present the results under three scenarios: the PVE of the gene expression level explained by ***β*** (the effect sizes of eQTLs) was equal to 0.3 or 0.5, the effect size *θ* (the effect size of the eQTL-based genetic score) was set to 0 or 0.4, and τ (the variance of the direct effect sizes of genetic variants) was set to 0 or 0.04. The results for other scenarios are displayed in Additional file 1: Figures S3-S9. As for the results shown in Fig. 2, we find the burden test is in general powerful when the association signal comes only from eQTL (i.e., *θ* = 0.4 and τ = 0), while is underpowered when the association signal comes only from SNPs (i.e., *θ* = 0 and τ = 0.04). The opposite results are observed for the KM test. Compared to the burden test and the KM test, the three joint tests (i.e., IEHC-Fisher, IEHC-adapt, and IEHC-optim) are often better when the association signal is contributed by both eQTL and SNPs (i.e., *θ* = 0.4 and τ = 0.04).

In addition, we find the relative performance of power between the joint tests (i.e., IEHC-Fisher, IEHC-adapt, and IEHC-optim) and the burden test as well as the KM test depends on the magnitude of *θ* and τ. More specifically, when *θ* and τ are not large enough, the burden test or (and) the KM test may behave better than the joint tests even the association signal is contributed by both the two components. For instance, the KM test is more powerful compared to the joint tests when *θ* = 0.1 and τ = 0.04 (Additional file 1: Figures S4); whereas the burden test has a higher power when *θ* = 0.2 and τ = 0.02 (Additional file 1: Figures S5). Finally, IEHC-ACAT, which integrates the five tests, consistently behaves better across various simulation scenarios (Fig. 2 and Additional file 1: Figures S3-S9).

### Correlation between *cis*-SNP marginal effect sizes of each gene in TCGA and GTEx

In the real application, we first quantify the association of *cis-*SNP effect sizes between TCGA and GTEx. To do so, for each SNP in TCGA we generated its marginal effect size using the general Cox model while adjusting for available cancer-specific covariates (e.g., age and tumor stage), and then conducted a simple linear regression with the two sets of estimated SNP effect sizes for each gene of these cancers. Note that, the SNP effect sizes of GTEx can be directly accessed through public portal (https://www.gtexportal.org/). Such regression analysis renders us a rough insight to interrogate the relationship of the two types of SNP effect sizes.

**Fig. 3:**
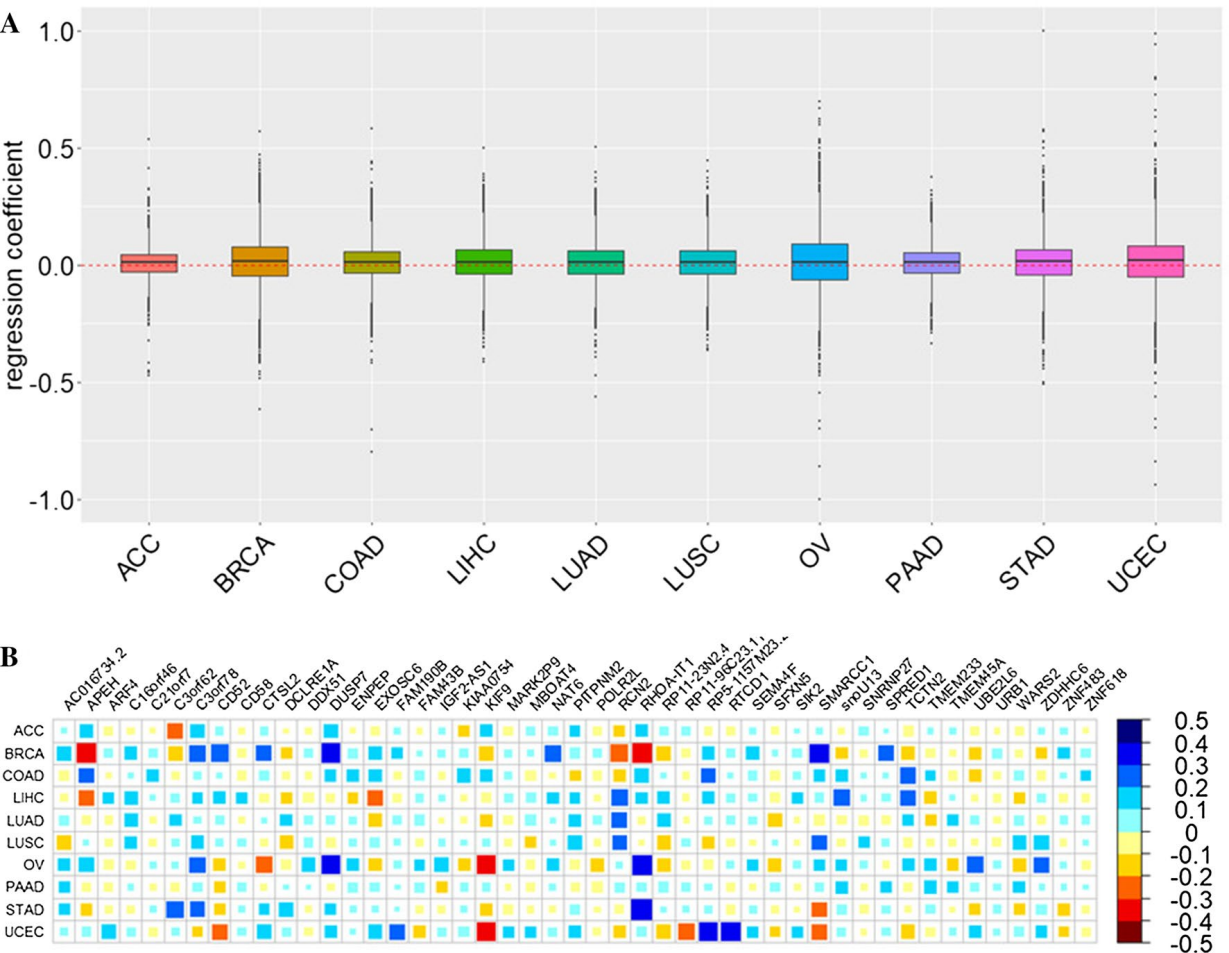
**A** Distribution of estimated regression coefficients for each gene across all the 10 TCGA cancers; **B** Heatmap of estimated regression coefficients of 47 of 118 genes that are simultaneously significant (FDR < 0.05) across all the 10 TCGA cancers; the density and the size of the color represent the magnitude of the regression coefficients

We discover that these two types of SNP effect sizes are substantially correlated for a great deal of genes for each cancer (Table 2). For example, we find that on average ~ 72.8% (ranging from 67.6% for BRCA to 76.4% for ACC) of regression coefficients are significant (false discover rate [FDR] < 0.05). Notably, for a given cancer the regression coefficients may be positive for some genes while negative for others (Fig. 3A). Particularly, among a total of 118 genes whose regression coefficients are significant across all the ten cancers, we still find the regression coefficients are either positive or negative across diverse cancers (Fig. 3B), indicating distinct genetic influences of SNPs on the regulation of gene expression and the survival risk of cancers. More importantly, a small fraction of (~ 3.4% on average) determination coefficients (*R*^2^) are larger than 10%, implying that the *cis*-SNP effect sizes of some certain genes in TCGA cancers can be indeed explained by eQTL of relevant tissue in the GTEx (Table 2).

**Table 2:** Summary information of *cis*-SNPs for the 10 cancers and the association of *cis*-SNP effect sizes for each gene in TCGA and GTEx

Cancer	Mean	Median	Max	Min	*R*^2^ > 0.10 (%)	FDR < 0.05 (%)
ACC	3,521	3,597	13,279	8	325 (2.9)	8533 (76.4)
BRCA	3,089	3,337	13,086	9	309 (4.0)	5265 (67.6)
COAD	3,655	3,737	12,881	4	297 (2.6)	8504 (73.7)
LIHC	3,492	3,597	8,479	3	202 (2.4)	6396 (74.6)
LUAD	3,732	3,733	12,717	20	170 (1.6)	7917 (74.6)
LUSC	3,525	3,611	12,726	6	282 (3.0)	7004 (74.2)
OV	1,935	1,750	6,752	3	977 (12.7)	5329 (69.0)
PAAD	3,801	3,784	13,382	9	167 (1.6)	7905 (73.7)
STAD	3,500	3,687	13,091	3	168 (1.8)	6496 (70.3)
UCEC	3,721	3,727	13,063	7	186 (1.7)	8086 (73.9)
Average	3,397	3,456	11,946	7	308 (3.4)	7145 (72.8)

Mean: the average number of *cis*-SNPs across genes; Max or Min: the maximal or minimal number of *cis*-SNPs across genes. *R*^2^ denotes the determination coefficient of the *cis*-SNP effect sizes for each gene in the linear regression with the effect sizes of a gene in TCGA as response and the effect sizes of that gene in GTEx as the covariate. The last column denotes the number of genes whose regression coefficient is significant (FDR < 0.05)

Taken together, although the average strength of the relationship between the two types of SNP effect sizes across genes may be relatively moderate, it nevertheless suggests potential genetic overlap especially at some certain genes. It is therefore worthy of integrating the eQTL of GTEx into the SNP-set based survival association analysis of TCGA cancers to boost the power.

### Associated genes identified with the IEHC method

**Fig. 4:**
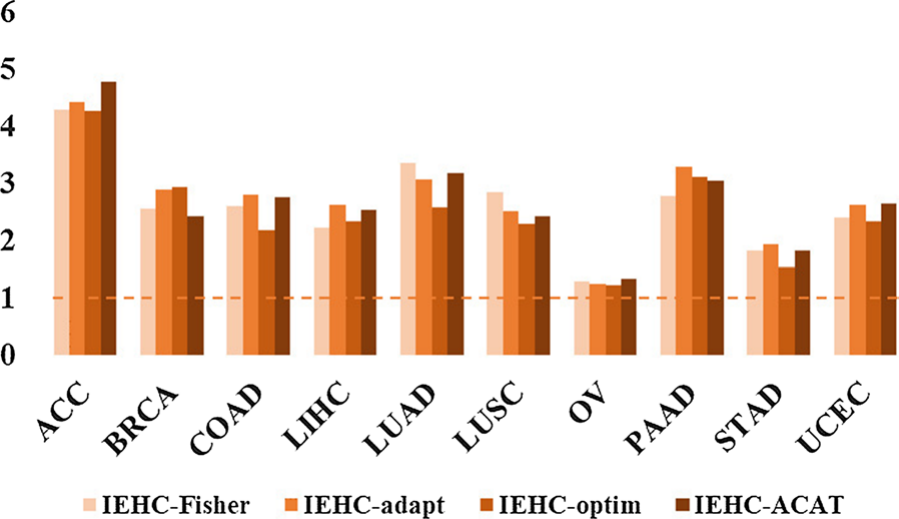
Improvement of the detection rate for genes with p-values of joint tests and the FDR of regression coefficients less than 0.05 across all the 10 TCGA cancers. Here the improvement is computed with the ratio of the detection rate for genes with significant regression coefficients and that for genes with non-significant regression coefficients. Thus, a ratio larger than one indicates the improvement

We here demonstrate that incorporating the eQTL information of GTEx into the SNP-set association analysis has the potential to enhance the power. We also exhibit that integrating all available tests by IEHC-ACAT can further increase the power. For each cancer and each type of joint tests (i.e., IEHC-Fisher, IEHC-optim, IEHC-adapt, and IEHC-ACAT), we classify all the genes into four various groups in terms of the regression coefficients (i.e., FDR < 0.05) and the results of joint tests (*P* < 0.05) (Additional file 1: Tables S1–S4 and Figures S10, S11). Taking ACC for example, there are a total of 8533 (= 259 + 8274) genes whose regression coefficients are significant (FDR < 0.05) and 2,630 (= 19 + 2611) genes whose regression coefficients are non-significant (FDR > 0.05); among these genes, 259 (~ 3.04% = 259/8533) and 19 (~ 0.72% = 19/2630) genes have a p-value less than 0.05 in terms of IEHC-Fisher, indicating that IEHC-Fisher has a fourfold higher likelihood (~ 4.22 = 3.04/0.72) to discover association signals (*p* = 4.61 × 10^–11^; Additional file 1: Table S1). The basic logic is that a smaller p-value would be generated in the joint tests if the eQTL of GTEx is predictive to the effect size of SNP in TCGA. Therefore, we expect that the detection rate of potentially associated genes (determined by *p* < 0.05) would be greater among genes with significant regression coefficients compared to those with non-significant regression coefficients. Formally, we employ the chi-square test to examine the difference in the detection rates (e.g., 3.04% vs. 0.72%) and observe a pronounced improvement of the detection rate for the four joint tests across nearly all cancers except OV (Fig. 4), in line with our expectation and suggesting the improvement of power when integrating the eQTL information of GTEx.

Finally, the number of associated genes identified with various test approaches is summarized in Table 3. Note that, the KM test cannot identify any associations, whereas a total of 21 (9 unique) genes are discovered for four cancers after incorporating the eQTL information of GTEx (Table 4). Specifically, IEHC-ACAT and the burden test detect 5 genes, followed by IEHC-adapt (4 genes), IEHC-optim (4 genes) and IEHC-Fisher (3 genes). We find that some genes are specifically discovered by some methods (e.g., *COL9A1*, *MSANTD2*, and *LMBRD1* by the burden test), although several genes are simultaneously identified by multiple tests (e.g., *RP11-1391J7.1* by the burden test, IEHC-adapt, IEHC-optim, and IEHC-ACAT), suggesting the various power of these test approaches across diverse genes, in line with the observation found in the simulations. Among the nine unique genes, the SNP effect sizes have a moderate correlation between TCGA and GTEx (Table 4 and Additional file 1: Figure S12).

**Table 3:** The number of significant genes identified by different test approaches in the 10 TCGA cancers (FDR < 0.1)

Cancer	Burden	KM	IEHC-Fisher	IEHC-adapt	IEHC-optim	IEHC-ACAT
BRCA	4	0	0	1	1	1
COAD	1	0	0	0	0	0
OV	0	0	2	2	3	3
STAD	0	0	1	1	0	1
Total	5	0	3	4	4	5

We here ignore those cancers for which non associated genes are discovered by any methods

**Table 4:** Summary information for associated genes for the four cancers identified by different tests

Cancer	Gene	chr (pos)	*S*	*r*	Burden	KM	IEHC-Fisher	IEHC-adapt	IEHC-optim	IEHC-ACAT
BRCA	*COL9A1*	6 (70,924,764–71,012,786)	4,121	− 0.32	*8.94* × *10*^*–2*^	8.43 × 10^–1^	1.41 × 10^–1^	1.54 × 10^–1^	2.18 × 10^–1^	1.56 × 10^–1^
BRCA	*MSANTD2*	11 (124,636,394–124,670,569)	4,563	− 0.16	*8.94* × *10*^*–2*^	8.43 × 10^–1^	4.64 × 10^–1^	2.03 × 10^–1^	2.20 × 10^–1^	2.33 × 10^–1^
BRCA	*LMBRD1*	6 (70,385,694–70,507,003)	4,324	0.26	*8.94* × *10*^*–2*^	8.43 × 10^–1^	1.67 × 10^–1^	1.54 × 10^–1^	2.18 × 10^–1^	1.56 × 10^–1^
BRCA	*RP11-1391J7.1*	11 (856,880–859,795)	4,309	0.34	*1.77* × *10*^*–2*^	8.43 × 10^–1^	1.25 × 10^–1^	*3.85* × *10*^*–2*^	*5.91* × *10*^*–2*^	*4.65* × *10*^*–2*^
COAD	*CTA-407F11.6*	22 (26,043,228–26,045,199)	4,290	0.23	*9.42* × *10*^*–2*^	8.41 × 10^–1^	6.67 × 10^–1^	2.09 × 10^–1^	3.04 × 10^–1^	2.48 × 10^–1^
OV	*KIAA1841*	2 (61,293,006–61,391,960)	3,162	0.01	9.52 × 10^–1^	2.62 × 10^–1^	*2.28* × *10*^*–4*^	*4.15* × *10*^*–5*^	*5.75* × *10*^*–2*^	*1.75* × *10*^*–4*^
OV	*FAM161A*	2 (62,051,989–62,081,278)	3,064	0.17	8.63 × 10^–1^	2.62 × 10^–1^	*1.70* × *10*^*–4*^	*4.15* × *10*^*–5*^	*1.68* × *10*^*–3*^	*1.75* × *10*^*–4*^
OV	*COMMD1*	2 (62,115,859–62,374,382)	3,047	0.16	9.93 × 10^–1^	2.26 × 10^–1^	6.75 × 10^–1^	5.38 × 10^–1^	*1.32* × *10*^*–2*^	*4.18* × *10*^*–2*^
STAD	*CTC-338M12.5*	5 (180,618,924–180,621,429)	1,051	0.06	9.96 × 10^–1^	5.88 × 10^–1^	*6.55* × *10*^*–4*^	*9.82* × *10*^*–5*^	4.63 × 10^–1^	*4.27* × *10*^*–4*^

The italic values represent genes identified by different tests (FDR < 0.1 is marked as italic)

*S*: the number of SNPs for each gene; *r*: estimated correlation coefficients for SNP effect sizes between the TCGA and GTEx datasets. The FDR level is set to 0.1

With regards to these discovered genes, there are previous studies which provided evidence supporting their associations with the cancers. For instance, it was discovered the methylation level of *COL9A1* reduced more evidently in tumors compared to that in the blood or healthy breast tissue, suggesting the association between *COL9A1* and the risk of breast cancer [48]. Dysregulation of *EGFR* expression and signaling was previously well documented to contribute to the progression and metastasis of breast cancer while *MSANTD2* played a crucial role in decreased epidermal growth factor endocytosis [49]. It was recently shown *LMBRD1* was significantly over-expressed in BRCA1 mutated cell line compared to BRCA1 wild-type cell line [50]. As another example, *COMMD1* was under-expressed in ovarian cancer, and the lack of detectable *COMMD1* protein expression was more frequent in ovarian cancer; *COMMD1* was also shown to be related to the cisplatin sensitivity in ovarian cancer [51]. In addition, we observe that four genes (i.e., *COL9A1*, *MSANTD2*, *LMBRD1* and *RP11-1391J7.1*) were differentially expressed between normal samples and tumor samples (Additional file 1: Figure S13), and that *COL9A1* was differentially expressed among different tumor stages (Additional file 1: Figure S14). In summary, these identified genes may represent potentially promising candidate biomarkers for cancer prediction, clinical treatment, and survival prognosis evaluation.

## Discussion

Recent technological advances in high-throughput platforms have greatly expanded the breadth of available omics datasets, including gene expression at the transcriptome level [36]. These abundant data resources facilitate to elucidate the interpretation of genetic variation in relation to survival risk and generate insightful perspective into the genetic underpinning of many complex human cancers [1–3]. However, how to effectively leverage the useful omics information is still an open problem. Therefore, there is a great demand for powerful analysis tools to fully harness the utility of these datasets. To fill such knowledge gap in the literature, herein we have proposed a novel genetic integrative Cox approach, called IEHC, to undertake the association analysis particularly for survival (time-to-event) phenotypes.

By characterizing effect sizes of SNPs between GTEx and TCGA, we found that there existed a substantial correlation across genes between the two types of effect sizes, indicating that we had the potential to improve the power if incorporating the GTEx eQTL into the survival SNP-set association studies. Methodologically, under the hierarchical model framework, IEHC has an appealing property that it models the effect sizes of SNPs as a function of variant characteristics (i.e., eQTL) to leverage information across loci while allowing for individual heterogeneous variant effects [26, 29]. Moreover, IEHC can be further interpreted within the framework of transcriptome-wide association studies (TWAS) [30, 31, 52]. In brief, the weighted burden score in IEHC (i.e., ${\text{G}}_{i}^{T} {\varvec{\beta}}$) is viewed as an imputed expression level with the weights of SNPs estimated from external tissue-related transcriptome reference datasets (i.e., GTEx), and the association between imputed expressions and cancers is examined for that gene while controlling for the direct effects of SNPs (i.e., ${\text{G}}_{i}^{T} {\varvec{b}}$). Because TWAS is effectively viewed as performing a two-sample causal inference [53, 54]; consequently, in this sense, IEHC has the ability to identify putative causal genes for cancers under certain regularity conditions [53–55].

Compared to the permutation test which is often computationally intensive, the proposed joint tests in IEHC are much more efficient because only two independent statistics are involved, both of which can be implemented with existing software and can be further combined via three kinds of p-values combination strategies. In addition, two previously used tests, including the burden test and the KM test, can be considered as special cases of the joint test. Furthermore, in IEHC we utilized ACAT to combine all these test methods. IEHC-ACAT enjoys the attractive strength that it takes the summary of a set of p-values as the test statistic and evaluates the significance analytically without the knowledge of correlation structure [34, 35]; thus, it is extraordinarily flexible and computationally fast. As a result, IEHC-ACAT allows us to aggregate dependent p-values obtained from these tests into a single well-calibrated p-value that can achieve the maximal power while maintaining the type I error correctly [34, 35, 56].

Extensive simulations revealed the relative performance of these joint test methods in IEHC and highlighted the strength of IEHC-ACAT. In agreement with the results of simulation, in the real application to ten TCGA cancers, we found that integrating eQTL can in general enhance the power and discover more genes that might be related to the survival risk of cancer. Particularly, IEHC-ACAT identified the highest number of associated genes among these competitive methods. In contrast, the KM test, which did not consider the eQTL of GTEx, cannot identify any association signals, suggesting the usefulness of integrating external informative variant annotations. Besides the attractive property in methodology, IEHC is also biologically interpretable when integrating transcriptomic information. First, it has been revealed that molecular features measured at the transcription level generally affect clinical outcomes more directly than those measured at other omic levels. Thus, the gene expression level would have the best predictive power for cancer prognostic evaluation compared to other genomic measurements [44]. Second, as it is widely demonstrated that SNPs associated with complex phenotypes are more likely to be eQTL [25], implying that gene expression may mediate the influence of genetic variants on the cancer risk. Therefore, eQTL can bridge the gap between cancers and many identified causal SNPs which have unknown function roles.

It needs to be emphasized that for the current IEHC model we only considered one type of variant characteristics (i.e., eQTL) but ignored other relevant information (e.g., protein quantitative trait loci). Therefore, the power of IEHC-Fisher, IEHC-adapt, IEHC-optim, and IEHC-ACAT may be further improved if more useful variant annotations would be employed in IEHC. The hierarchical modeling in IEHC offers an effective and general manner to incorporate more functional annotations as they become available. However, when many functional annotations can be applied, some of them may not be useful for determining associated genes. Therefore, the selection of informative annotations during the association analysis is necessary, which may be an interesting avenue for future investigations. Furthermore, although there include many cancer types in TCGA, their effective sample sizes are still relatively small, and the censored proportions are high [43, 44], which inevitably undermines the power of any methods and may lead to the failure of identifying some associated genes with survival. In addition, we only considered the linear kernel in these joint tests of IEHC when assessing the direct effects of SNPs (i.e., *H*_0_: τ = 0). The linear kernel may be sub-optimal if the relationship between SNPs and the survival risk is non-linear. Intuitively, the power of IEHC would depends on how well the chosen kernel captures the true relationship between SNPs and the survival risk, which can differ in the numbers, effect sizes, and effect directions of causal variants across diverse genes. For example, if only a very small fraction of SNPs may be causal, then the sparse kernel should be a better choice; if SNPs have mutual interaction effect sizes, then the product kernel consisting of main effects and interaction terms is preferred. However, in practice the relation is rarely known in advance, selecting an optimal kernel may be very challenging [20, 57–61]. Therefore, adaptive IEHC model and test methods for multiple candidate kernel functions are warranted to study in the future [20].

## Conclusion

Overall, IEHC represents a flexible, robust, and powerful approach to integrate functionally omic datasets to improve the power of identifying associated genes for the survival risk of complex human cancers.

## Supplementary Information

**Additional file 1.**

## Data Availability

All data generated or analyzed during this study are included in this published article and its additional file.

## Abbreviations

GWAS: Genome-wide association study
ACAT: Aggregated Cauchy association test
eQTL: Expression quantitative trait loci
SNP: Single nucleotide polymorphisms
GTEx: Genotype-Tissue Expression
FDR: False discover rate
TWAS: Transcriptome-wide association study
IEHC: Integrative eQTL hierarchical Cox model
