# Supplementary material for: Integrative eQTL-weighted hierarchical Cox models for SNP-set based time-to-event association studies
Source: J Transl Med. 2021 Oct 9;19:418. doi: 10.1186/s12967-021-03090-z (PMC8502405; doi:10.1186/s12967-021-03090-z)
Supplement: Supplementary file 1 — Additional file 1. [file 12967_2021_3090_MOESM1_ESM.docx]

**Supplementary File**

### Mixed-effects Cox model with multiple SNPs

Consider that there are *S* genotypes (denoted by G*_i_* and coded as 0, 1 or 2 in terms of the number of effect allele) of SNPs located within a given gene and *p* covariates X*_i_* (e.g., age, gender, and cancer stage) for *n* individuals; and *S* varies gene by gene. In addition, denote the observed survival time by *t_i_* and the true survival time by *T_i_* with *d_i_* indicating the censored status; that is, *d_i_* = 1 if *T_i_* = *t_i_*, whereas *d_i_* = 0 if *T_i_* < *t_i_*. under the proportional hazards condition, we assume the hazard function λ(*t*) of the survival time *t_i_* is related to G*_i_* and X*_i_* through the classical Cox model [[1](#_ENREF_1)]

$$\log(\frac{\text{λ}(t_{i})}{\text{λ}_{0}(t_{i})}) = \text{G}_{i}^{T}\alpha\text{+} \text{X}_{i}^{T}c$$

where λ_0_ is an arbitrary baseline hazard function, ***α*** = (*α*_1_, …, *α_S_*) is an *S*-vector of effect sizes for SNPs and ***c*** = (*c*_1_, *c*_2_, …, *c_p_*) is a *p*-vector of fixed effect sizes for clinical covariates.

Due to the number of SNPs included within a gene may be rather large (sometimes, even *S* > *n*), to avoid the issue of un-identifiability and leverage genetic information across diverse SNPs, we thus treat ***α*** = (*α*_1_, …, *α_S_*) as random effect following a normal distribution *α_j_* ~ *N*(0, τ), leading to the so-called Cox linear mixed-effects model (coxlmm) [[2](#_ENREF_2)]. An alternative strategy is to treat ***α*** as fixed effect and perform the likelihood ratio test or the score test with *S* degrees of freedom. However, these two types of tests are subject to great power loss in high-dimensional hypothesis. Moreover, if only a small subset of SNPs are associated with the survival risk, testing for τ = 0 is often more powerful [[3](#_ENREF_3)]. Of note, model can be also viewed as a special case of kernel machine (KM) survival models with a linear kernel function [[4](#_ENREF_4), [5](#_ENREF_5)]. Within the framework of model , we examine the following null hypothesis to determine whether a set of SNPs are collectively associated with the survival risk

$$H_{0}: \alpha_{1} = \cdots= \alpha_{S} = 0 \Leftrightarrow H_{0}: \text{τ} = 0$$

Model can still lose power when some knowledge is available about the alternative (i.e., τ ≠ 0 or not all *α_j_*’s are zero). For example, if the association between a set of genetic variants and the survival risk of cancers is regulated through gene expression, the power gain can be achieved by integrating transcriptome information into the test. As it is widely demonstrated that associated SNPs are more likely to be expression quantitative trait loci (eQTL) [[6](#_ENREF_6)], it is thus conceivable that incorporating such knowledge would improve the power for detecting association [[7](#_ENREF_7)].

### Hierarchical model to integrate eQTL information

To integrate existing eQTL information, such as GTEx [[8](#_ENREF_8), [9](#_ENREF_9)], into model , we propose a hierarchical regression to model ***α*** as a response variable and eQTLs as covariates. Specifically, we suppose that ***β*** = (*β*_1_, …, *β_S_*)*^T^* is an *S*-vector of known eQTL effect sizes of the *S* SNPs, which can be directly obtained from GTEx summary statistics, and ***α*** is in part explained by ***β***

$$\alpha_{j} = \beta_{j} \times\theta+ b_{j}, j = 1, ..., S$$

where *θ* is a scale of coefficient for eQTL and *b* is the residual variant-specific effect size that is not interpreted by eQTL alone. Like before, we assume *b* follows a normal distribution. Plugging into model , we obtain the hierarchical Cox model

$$\begin{matrix} \log(\frac{\text{λ}(t_{i})}{\text{λ}_{0}(t_{i})}) = (\text{G}_{i}^{T}\beta) \times\theta\text{+} \text{G}_{i}^{T}b \text{+} \text{X}_{i}^{T}c, \text{ } \\ b \sim N(0, \text{τ}) \end{matrix}$$

We refer to as the integrative eQTL hierarchical Cox (IEHC) model. Within the framework of IEHC, *θ* quantifies the association between the survival risk and the weighted burden score $\text{G}_{i}^{T}\beta$. Now the hypothesis of no association becomes

$$H_{0}: \theta= 0 \text{and} b = 0 \Leftrightarrow H_{0}: \theta= 0 \text{and} \text{τ} = 0$$

This is a joint test including both fixed effect and random effects: the first component of *H*_0_ examines the influence of genetic variants on the survival risk explained by eQTLs; while the second component examines the impact of genetic variants beyond the effects of eQTLs.

### Framework of joint test of fixed effect and random effects in IEHC

To jointly examine *H*_0_ given in , we can attempt to employ a score test to assess the joint effects of SNPs on the survival outcome within the framework of KM Cox regression [[4](#_ENREF_4), [5](#_ENREF_5)], because the score test avoids estimating the variance component τ under the alternative, which is generally not easy due to the high-dimensional integration [[2](#_ENREF_2), [10](#_ENREF_10)]. Then, the respective statistics for *θ* and τ can be obtained under the null, with the statistic for *θ* following a normal distribution and the statistic for τ following a mixture of chi-squared distribution. However, these two statistics are correlated; it is hence theoretically difficult to derive their joint null distribution.

To circumvent this problem, following previous work we propose to modify the two statistics so that they are independent [[3](#_ENREF_3), [7](#_ENREF_7)]. Briefly, we derive the test statistic for *θ* under *H*_0_: *θ =* 0 and τ = 0 as usual, while we derive the score statistic for τ under τ = 0 but without the constraint of *θ* = 0. By doing this, we ensure that these two statistics are independent (Figure S1). This strategy substantially eases the development of test statistics for the joint test shown in . In conclusion, under this framework two asymptotically independent statistics can be derived: one for a scale (i.e., *θ*) in the general Cox model [[1](#_ENREF_1)] and the other for the variance component (i.e., τ) in the KM Cox model [[4](#_ENREF_4), [5](#_ENREF_5)]. The detailed procedures for this joint test in IEHC are illustrated as follows.

#### The Burden test for examining fixed effect in the general Cox model

Testing *H*_0_: *θ* = 0 is equivalent to examining whether there is a burden effect of the genetic variants explained by eQTL [[11](#_ENREF_11), [12](#_ENREF_12)]. We derive the burden statistic for *θ* in IEHC under *H*_0_: *θ* = 0 and τ = 0, and have the following reduced Cox model

$$\log(\frac{\text{λ}(t)}{\text{λ}_{0}(t)}) = (\text{G}_{i}^{T}\beta) \times\theta\text{+} \text{X}_{i}^{T}c$$

We can easily obtain the score statistic (denoted by *U_θ_*) as well as its corresponding p-value (denoted by *P_θ_*). The test of *θ* is implemented with the coxph function in R.

#### Testing variance component in the kernel machine Cox model

Testing *H*_0_: τ = 0 is equivalent to examining whether there is a main joint effect of genetic variants on the survival risk not explained by eQTL. We derive the score statistic for τ in IEHC under τ = 0 but without constraining *θ* = 0. Under such a random-effects framework, we obtain the corresponding score statistic (denoted by *U_τ_*) of variance component in the KM Cox model [[4](#_ENREF_4), [5](#_ENREF_5)], which follows a mixture of chi-squared distribution and its p-values (denoted by *P_τ_*) can be yielded by the coxKM package in R [[4](#_ENREF_4), [5](#_ENREF_5)].

#### Numerical consideration for p-value calculation in the KM Cox models

Although it has been shown that the KM test statistic *U_τ_* follows a mixture of χ2 1 under the null, there lacks an analytical form for this mixture distribution. In order to calculate the p-value quickly without resorting to Monte Carlo simulation, various numerical algorithms have been proposed to calculate the cumulative probability distribution. These include the Satterthwaite approximation [[13](#_ENREF_13)], the Davies method [[14](#_ENREF_14)], and the moment matching method (i.e., the Liu’s method) [[15](#_ENREF_15)]. The Davies method involves inverting the characteristic function of the mixture distribution to the corresponding cumulative distribution function, and then utilizes the numerical integration to calculate the p-value. The Liu’s method approximates the mixture distribution by a non-central χ^2^ distribution that matches on the third moment and minimizes the difference of the fourth moment with the mixture distribution. Recently, the Liu’s method has also been modified to match on the fourth moment while minimizing the difference of the third moment; and it thus offers more accurate approximation on extreme tail probabilities [[16](#_ENREF_16)].

#### Joint tests for combining fixed- and random-effects components

It can be imagined that the power of the combination tests based on individual components alone is highly sensitive to the relative contribution of signals from the two components. The weighted linear summation of the two statistics is perhaps the most straightforward combination approach. We first utilize the famous Fisher’s combination approach for aggregating the two independent test statistics [[17](#_ENREF_17), [18](#_ENREF_18)], which simply takes the summation of -2log(p-value) from individual tests as the measurement of the overall significance. The combined joint test statistic follows a chi-squared distribution with four degrees of freedom due to the independence. Although the Fisher’s method gains power when both *θ* (i.e., eQTL) and τ (i.e., SNPs) contribute to the association, it can lose power if only one of the two components shows association. This motivates us to develop alternative weighted combination methods so that the weight can better reflect the signal that comes from a particular component if only one of the two components is associated with the survival risk. In the following we introduce another two data-driven weighted combination strategies including optimally weighted linear combination (optim) and adaptively weighted linear combination (adapt).

#### An optimally weighted linear combination

In the optimally weighted linear combination, we construct *T_ρ_* = *ρ**U_θ_* + (1-*ρ*)*U_τ_*, where $\rho\in[0, 1]$ controls the contribution of the fixed-effect component. An intuitive approach to determine the optimal weight *ρ*^*^ is to minimize the p-value *p_ρ_* based on *T_ρ_*, i.e., $\rho^{*} = \mathop{\mathrm{argmin}}\limits_{\rho\in[0, 1]} \{p_{\rho}\}$. Although there is no analytical form of *ρ*^*^ due to the complex expression of *p_ρ_*, various numerical optimization techniques can be applied to find *ρ*^*^. We denote the observed minimal p-value as *Pobs ρ**, with *ρ*^*^ the optimal weight. To account for the fact that the minimal p-value is used, we calculate the p-value

$$\begin{matrix} \Pr(P_{\rho^{*}} \leq P_{\rho^{*}}^{obs}) = 1 - \Pr(P_{\rho} \geq P_{\rho^{*}}^{obs}, \forall\rho\in[0, 1]) \\ \text{=} 1 - \text{E}\left\{\text{I}\left(U_{\theta} < Q_{U_{\theta}}(1 - P_{\rho^{*}}^{obs})\right)\right. \\ \left.\times\Pr\left(U_{\tau} < \min_{\rho\neq1} \frac{1}{1 - \rho}[Q_{T_{\rho}}(1 - P_{\rho^{*}}^{obs}) - \rho U_{\theta}]|U_{\theta}\right)\right\} \end{matrix}$$

where *Q_U_*(*p*) is the (100*p*)^th^ quantile of the uniform random variable *U* and the expectation is evaluated with respect to *U_θ_*. For a given *ρ*, the quantile $Q_{T_{\rho}}(1 - P_{\rho^{*}}^{obs})$ also has no analytical form; hence numerical approximations are applied. In addition, the expectation in can be obtained by fast numerical integration methods.

#### An adaptively weighted linear combination

The adaptively weighted linear combination is a data-adaptive generalization of the Fisher’s combination [[17](#_ENREF_17), [18](#_ENREF_18)]. The test statistic takes the form *T* = *ρ_θ_Z_θ_* + *ρ_τ_**Z_τ_*, where *Z_θ_* = -2log(*p_θ_*) and *Z_τ_* = -2log(*p_τ_*). Under this combination we acquire *ρ_θ_* and *ρ_τ_* determined by *Z_θ_* and *Z_τ_* via the following two formulas

$$\rho_{\theta} = \frac{Z_{\theta}}{\sqrt{Z_{\theta}^{2} + Z_{\tau}^{2}}} \text{and} \rho_{\tau} = \frac{Z_{\tau}}{\sqrt{Z_{\theta}^{2} + Z_{\tau}^{2}}}$$

Intuitively, the two weights are equivalent to the sine and cosine functions of the angle between the direction of the observed $(Z_{\theta}, Z_{\tau}) \in\text{R}^{2}$ and the x*-*axis. The test statistic can be further simplified and expressed as *T* = *Z2 θ* + *Z2 τ*. This simplified form of *T* provides an insight on its asymptotic null distribution, which is the summation of squares of two independent χ2 2 distributions. The numerical integration approaches can be employed in the p-value calculation at a low computational cost.


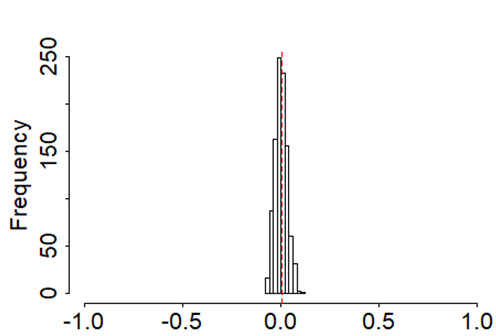


Figure S1. Histogram of the correlation coefficients between *U_θ_* and *U_τ_*. A total of 10^5^ pairs of *U_θ_* and *U_τ_* were generated under the null in our simulation. The overall correlation coefficient between *U_θ_* and *U_τ_* is 1.75×10^-3^ (95% confidence interval [CI] -4.44×10^-3^ - 7.95×10^-3^, *P* = 0.580). Furthermore, we randomly sampled 10^3^ pairs of *U_θ_* and *U_τ_* without replacement, and calculated the correlation coefficient. We repeated this calculation 10^3^ times and produced this histogram. It is shown that the correlation coefficient ranges from -0.1 to 0.1, indicating the independence between *U_θ_* and *U_τ_*.


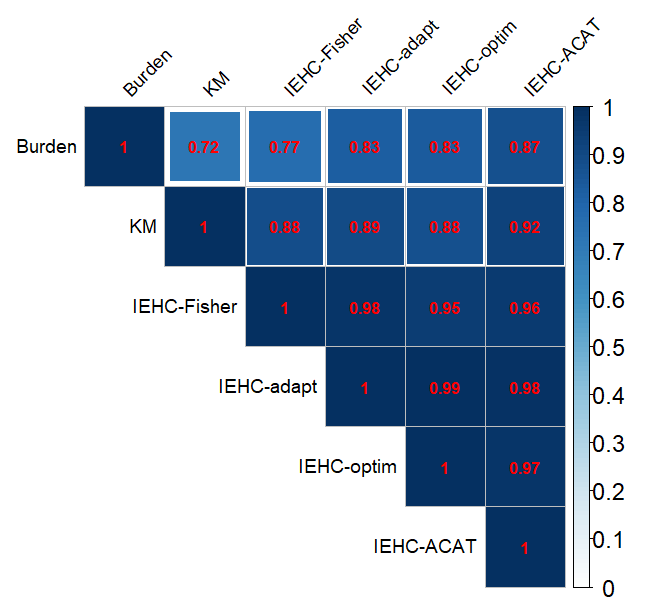


Figure S2. Correlation of -log10(p-value) under the null that both *θ* and τ are zero in the IEHC model for the six methods including the burden test, the KM test, IEHC-Fisher, IEHC-adapt, IEHC-optim as well as IEHC-ACAT.


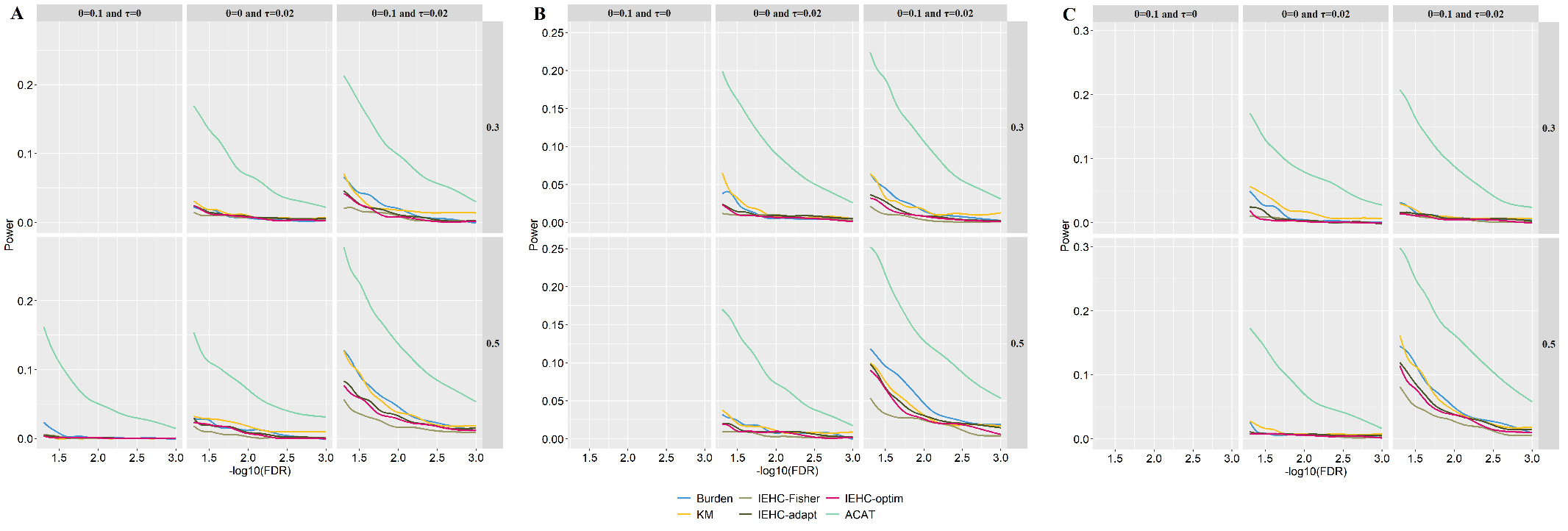


Figure S3. Power comparison among the six methods under the alternative. In the simulation scenarios, 30%, 50% or 0% SNPs were randomly selected to have zero effect sizes. The PVE of the expression level explained by ***β*** was set to 0.3 (above) or 0.5 (below). (A) 30% SNPs having zero effect sizes; (B) 50% SNPs having zero effect sizes; (C) 0% SNPs having zero effect sizes. Here, *θ* = 0.1 or (and) τ = 0.02.


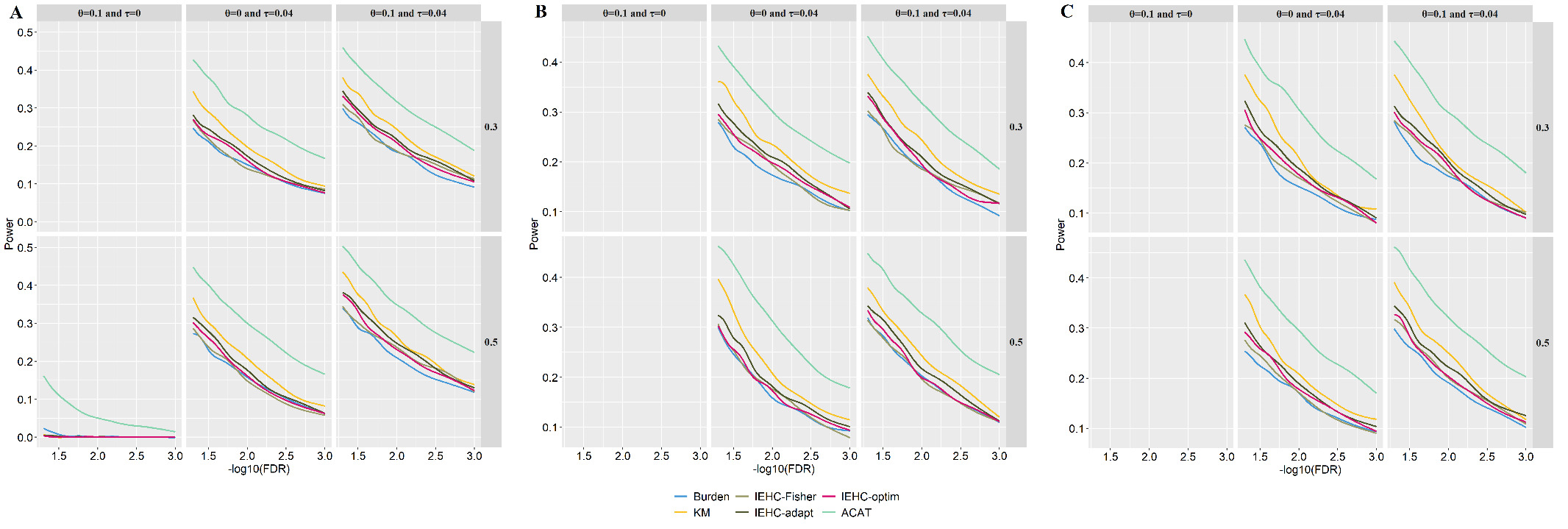


Figure S4. Power comparison among the six methods under the alternative. In the simulation scenarios, 30%, 50% or 0% SNPs were randomly selected to have zero effect sizes. The PVE of the expression level explained by ***β*** was set to 0.3 (above) or 0.5 (below). (A) 30% SNPs having zero effect sizes; (B) 50% SNPs having zero effect sizes; (C) 0% SNPs having zero effect sizes. Here, *θ* = 0.1 or (and) τ = 0.04.


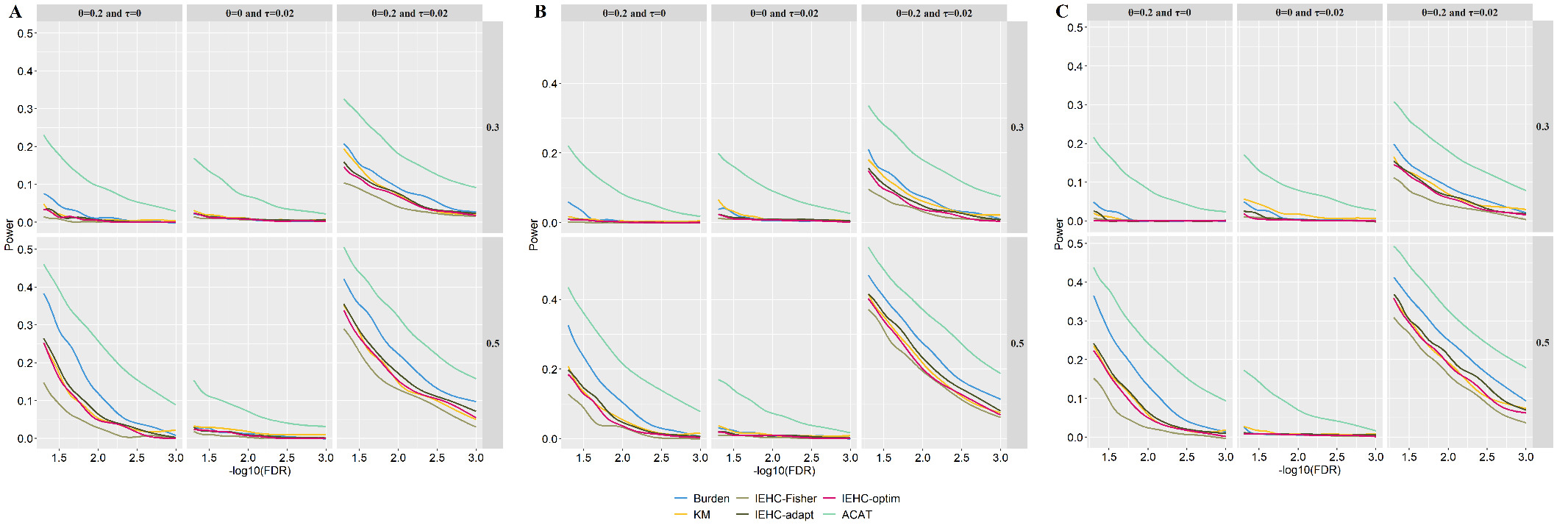


Figure S5. Power comparison among the six methods under the alternative. In the simulation scenarios, 30%, 50% or 0% SNPs were randomly selected to have zero effect sizes. The PVE of the expression level explained by ***β*** was set to 0.3 (above) or 0.5 (below). (A) 30% SNPs having zero effect sizes; (B) 50% SNPs having zero effect sizes; (C) 0% SNPs having zero effect sizes. Here, *θ* = 0.2 or (and) τ = 0.02.


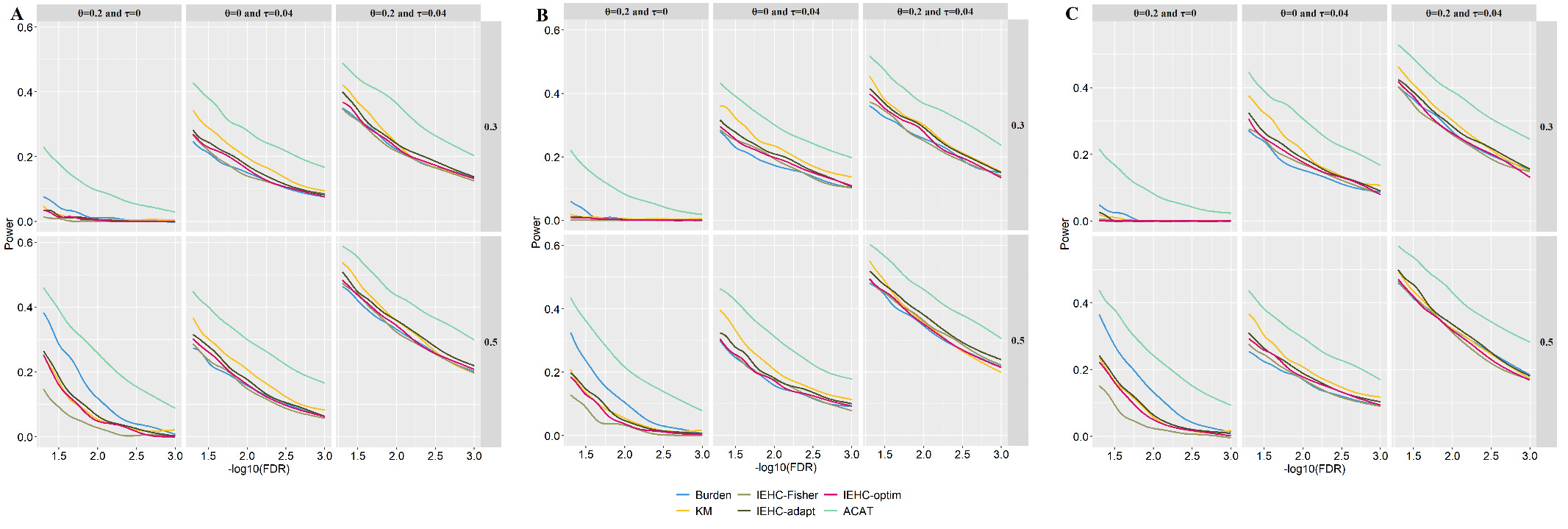


Figure S6. Power comparison among the six methods under the alternative. In the simulation scenarios, 30%, 50% or 0% SNPs were randomly selected to have zero effect sizes. The PVE of the expression level explained by ***β*** was set to 0.3 (above) or 0.5 (below). (A) 30% SNPs having zero effect sizes; (B) 50% SNPs having zero effect sizes; (C) 0% SNPs having zero effect sizes. Here, *θ* = 0.2 or (and) τ = 0.04.


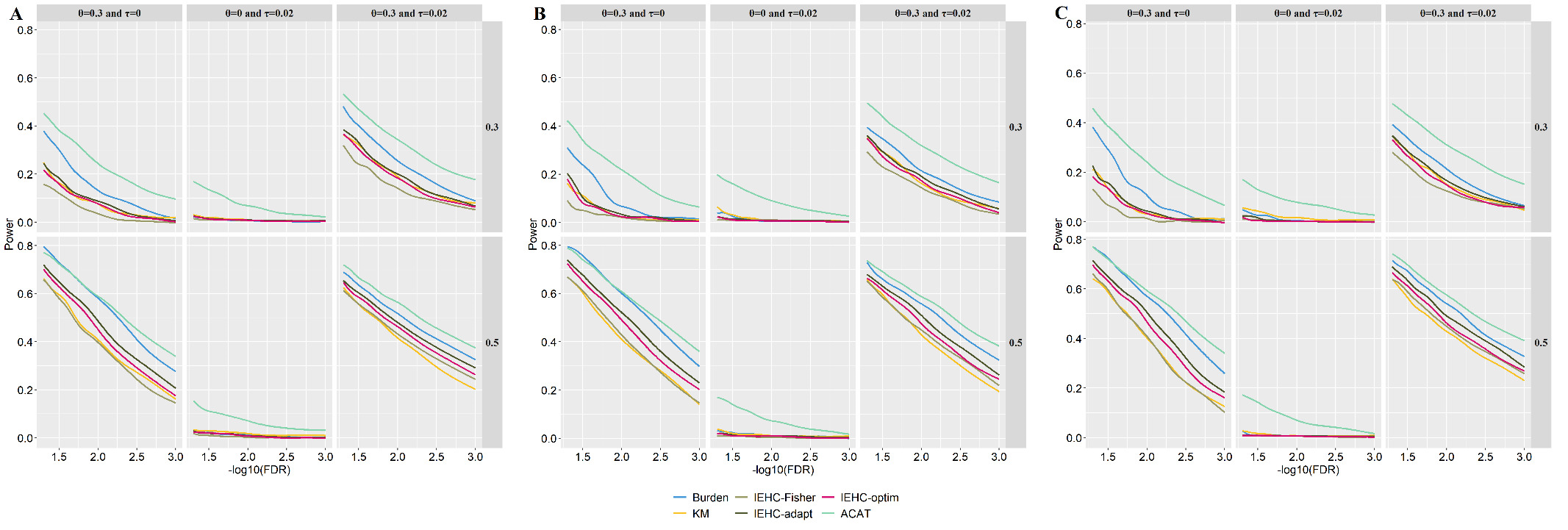


Figure S7. Power comparison among the six methods under the alternative. In the simulation scenarios, 30%, 50% or 0% SNPs were randomly selected to have zero effect sizes. The PVE of the expression level explained by ***β*** was set to 0.3 (above) or 0.5 (below). (A) 30% SNPs having zero effect sizes; (B) 50% SNPs having zero effect sizes; (C) 0% SNPs having zero effect sizes. Here, *θ* = 0.3 or (and) τ = 0.02.


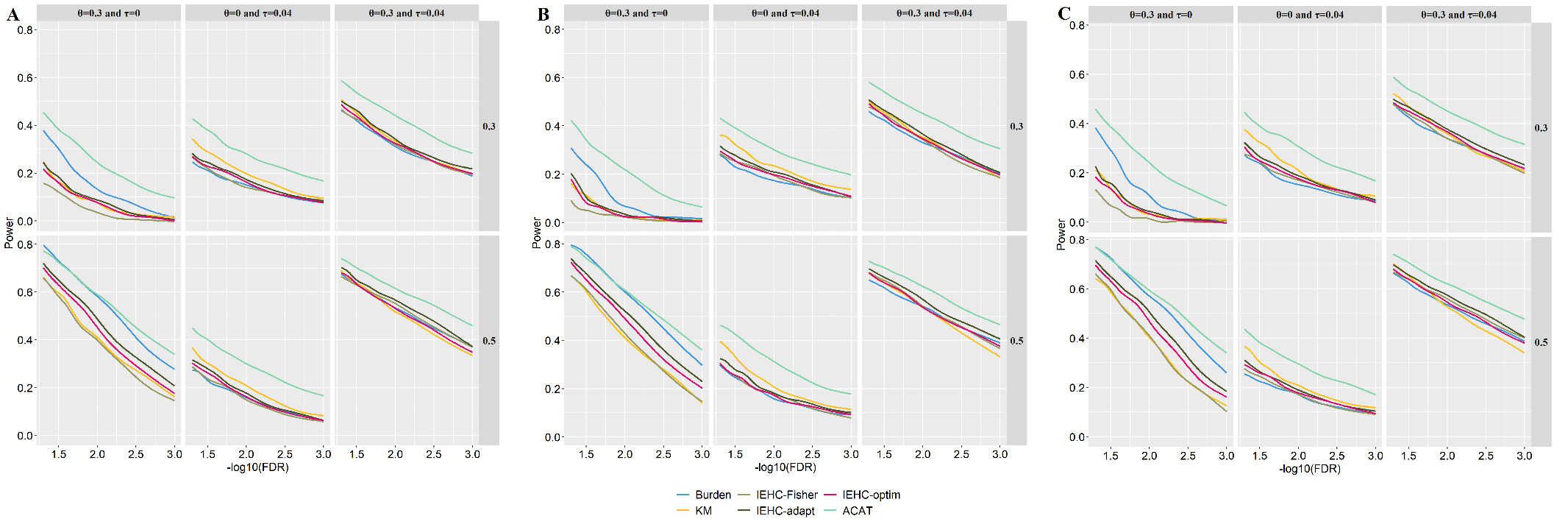


Figure S8. Power comparison among the six methods under the alternative. In the simulation scenarios, 30%, 50% or 0% SNPs were randomly selected to have zero effect sizes. The PVE of the expression level explained by ***β*** was set to 0.3 (above) or 0.5 (below). (A) 30% SNPs having zero effect sizes; (B) 50% SNPs having zero effect sizes; (C) 0% SNPs having zero effect sizes. Here, *θ* = 0.3 or (and) τ = 0.04.


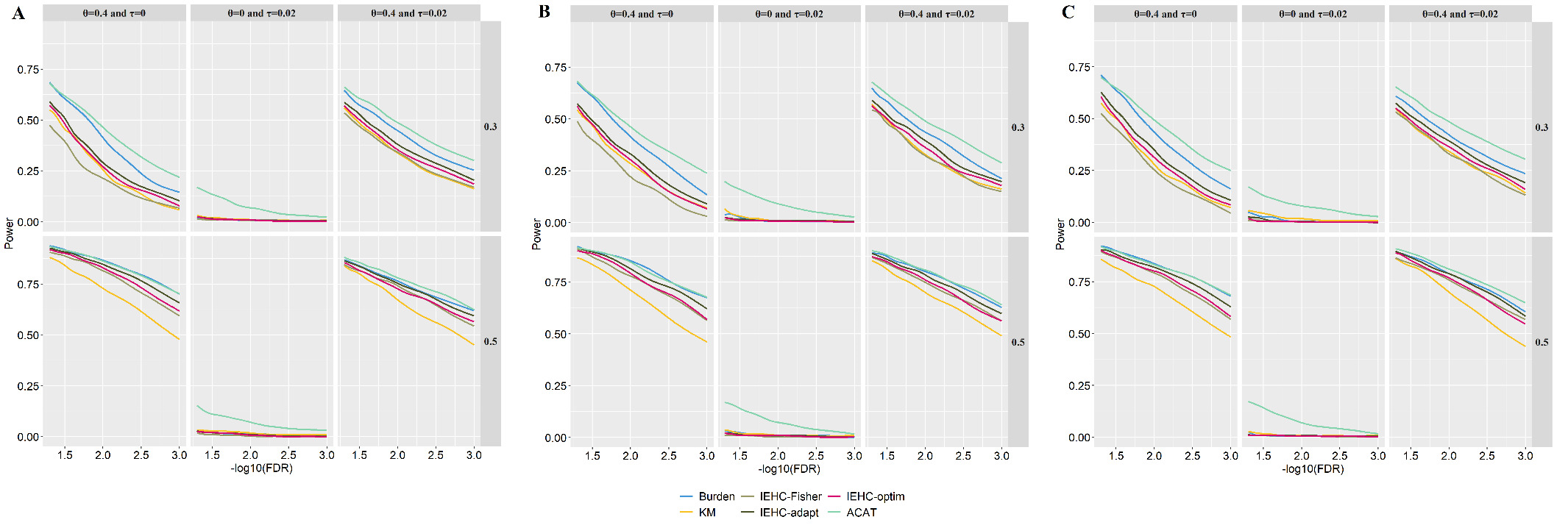


Figure S9. Power comparison among the six methods under the alternative. In the simulation scenarios, 30%, 50% or 0% SNPs were randomly selected to have zero effect sizes. The PVE of the expression level explained by ***β*** was set to 0.3 (above) or 0.5 (below). (A) 30% SNPs having zero effect sizes; (B) 50% SNPs having zero effect sizes; (C) 0% SNPs having zero effect sizes. Here, *θ* = 0.4 or (and) τ = 0.02.


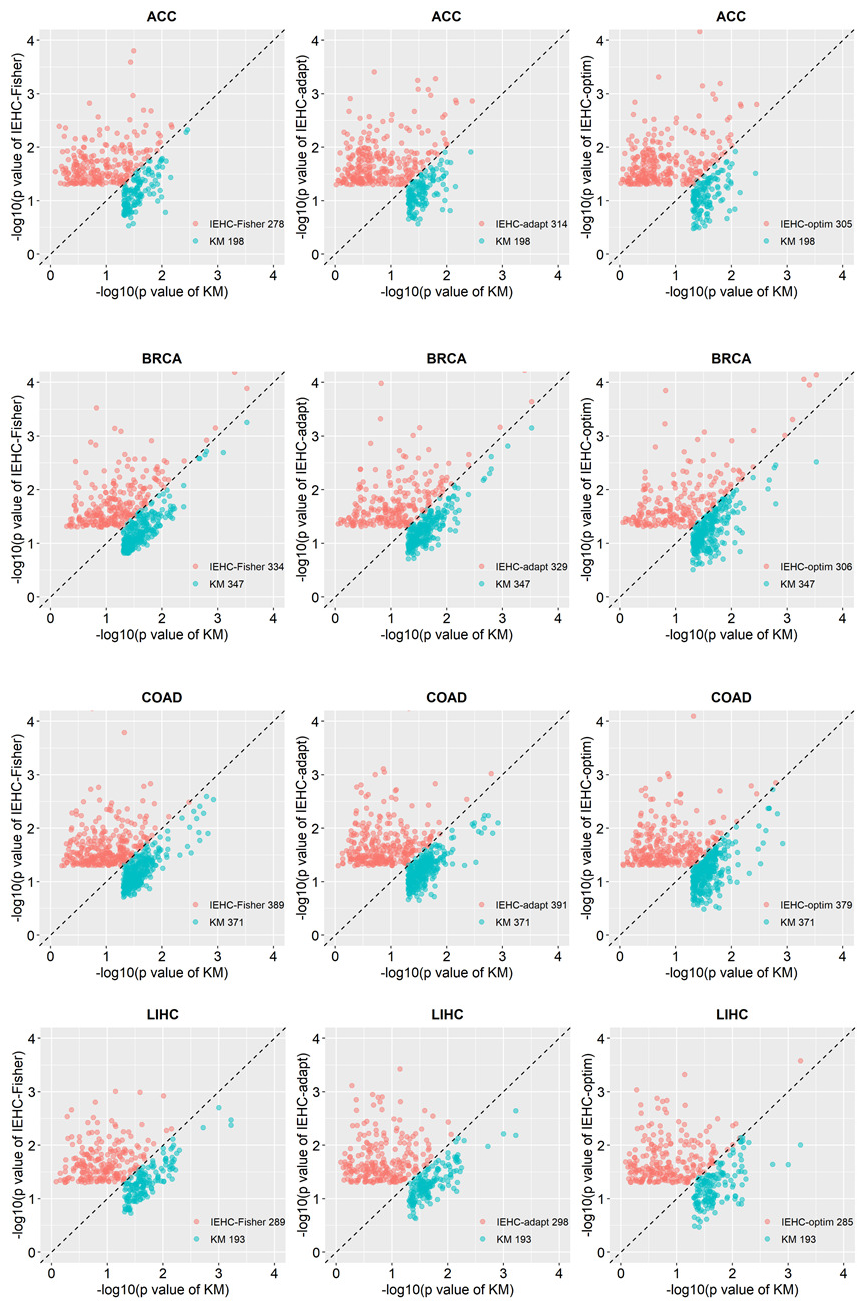


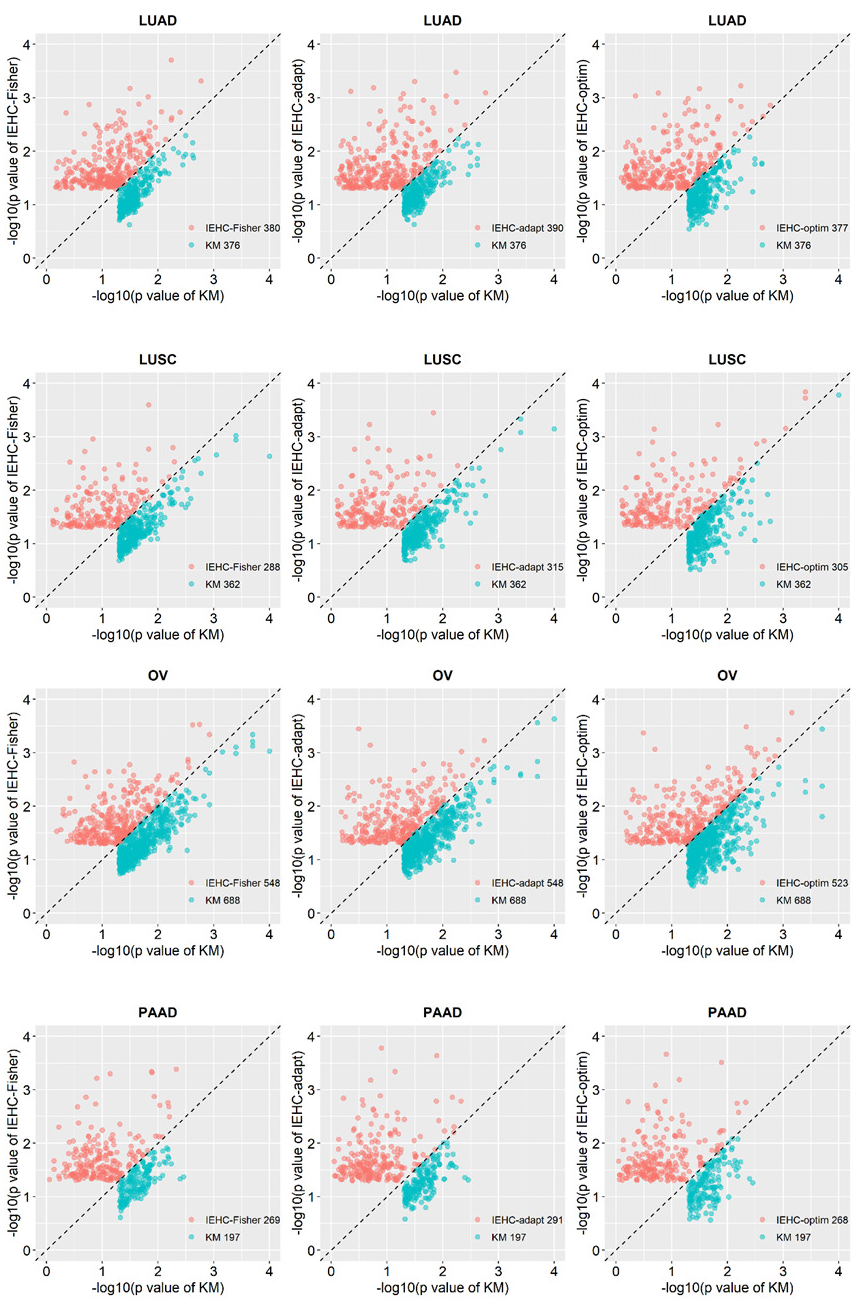


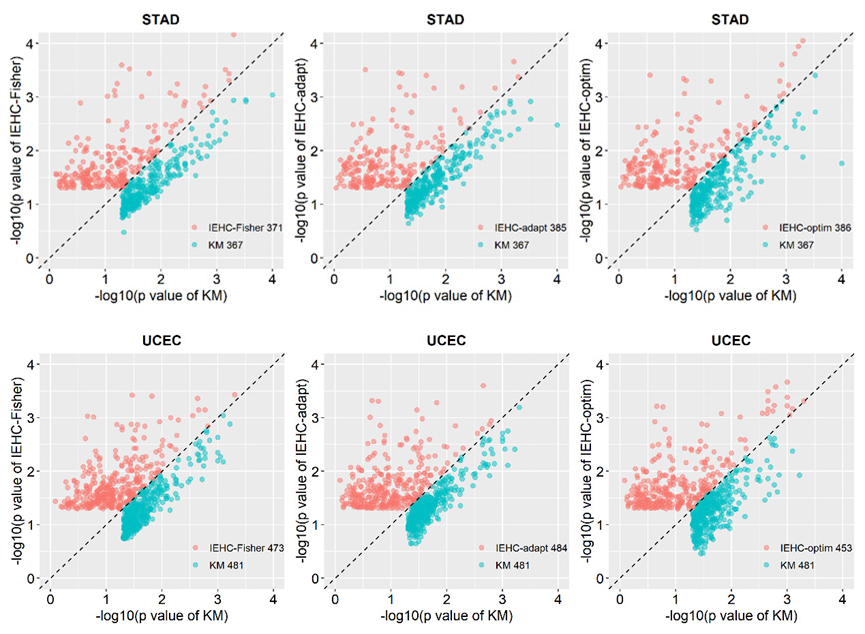


Figure S10. Scatter plots of p-values for the three joint test methods and the KM test for the 10 cancers. The number of genes whose p-values are smaller than 0.05 is shown.


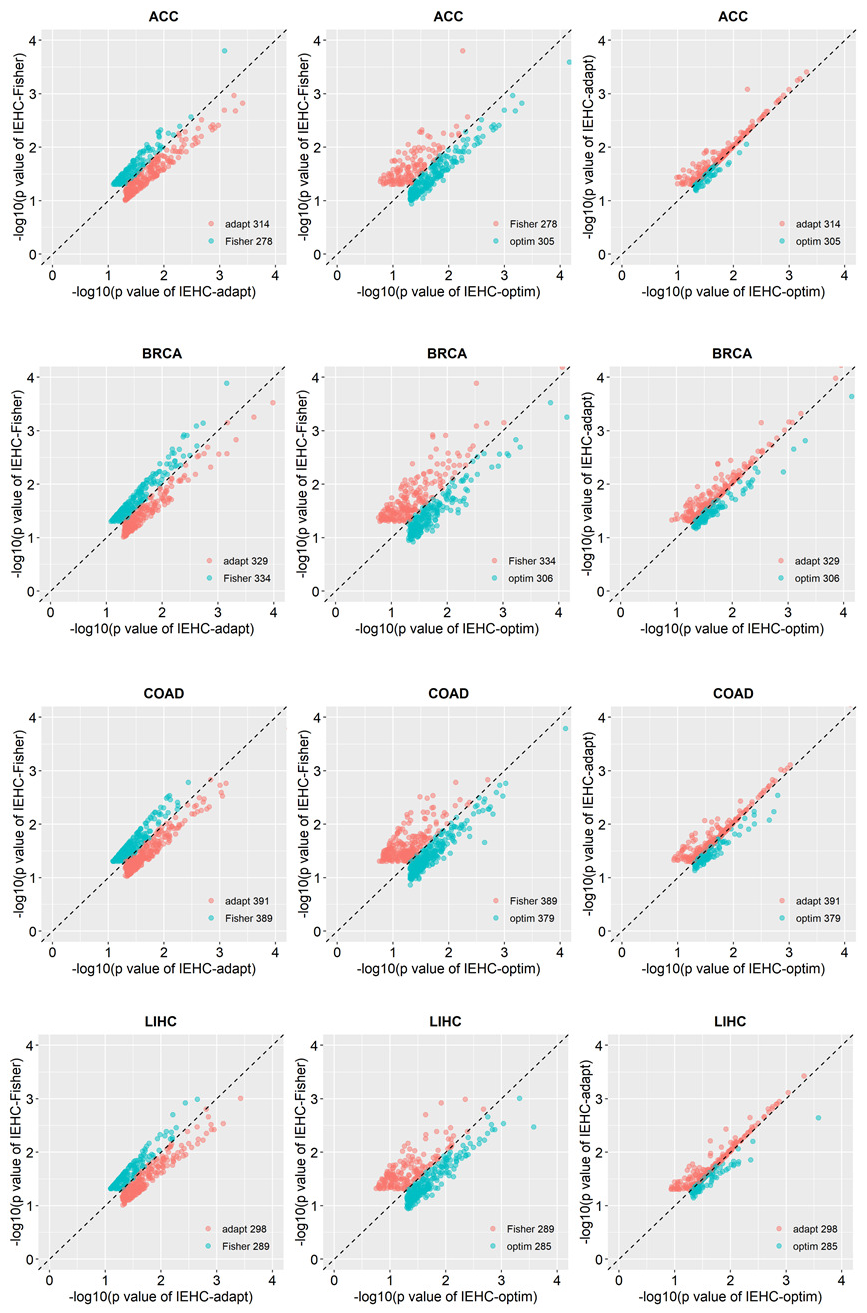


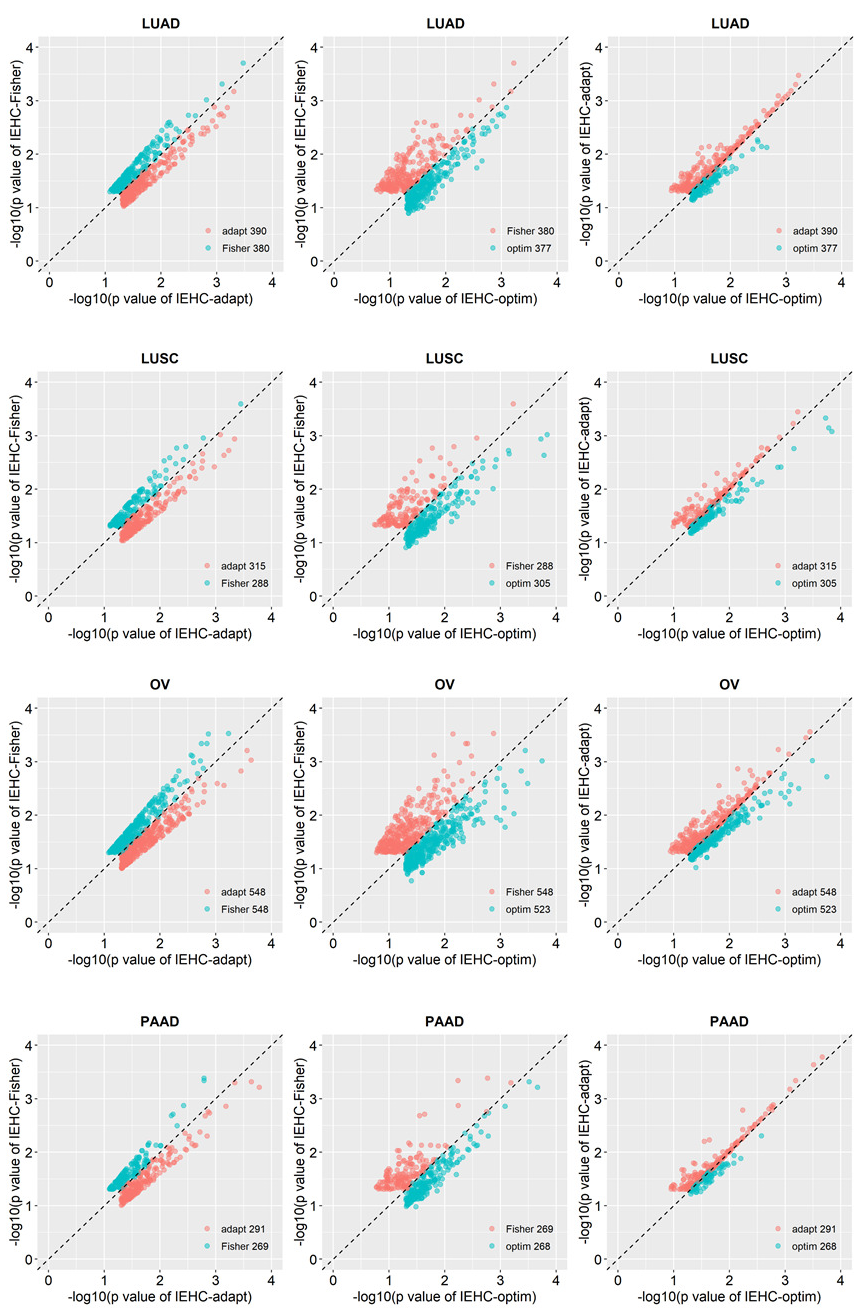


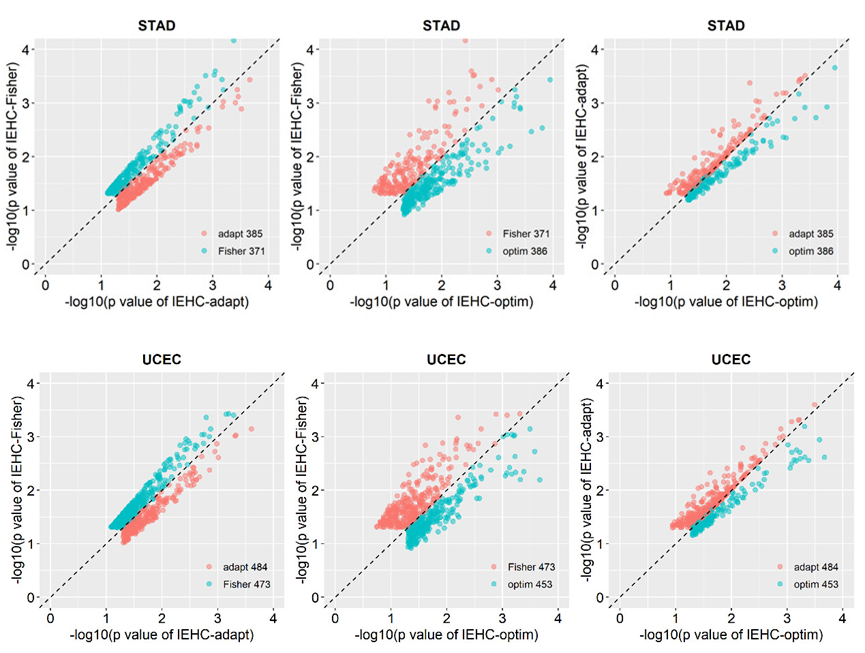


Figure S11. Scatter plots of p-values of the three joint test methods for 10 cancers. The number of genes whose p*-*values are smaller than 0.05 is shown.


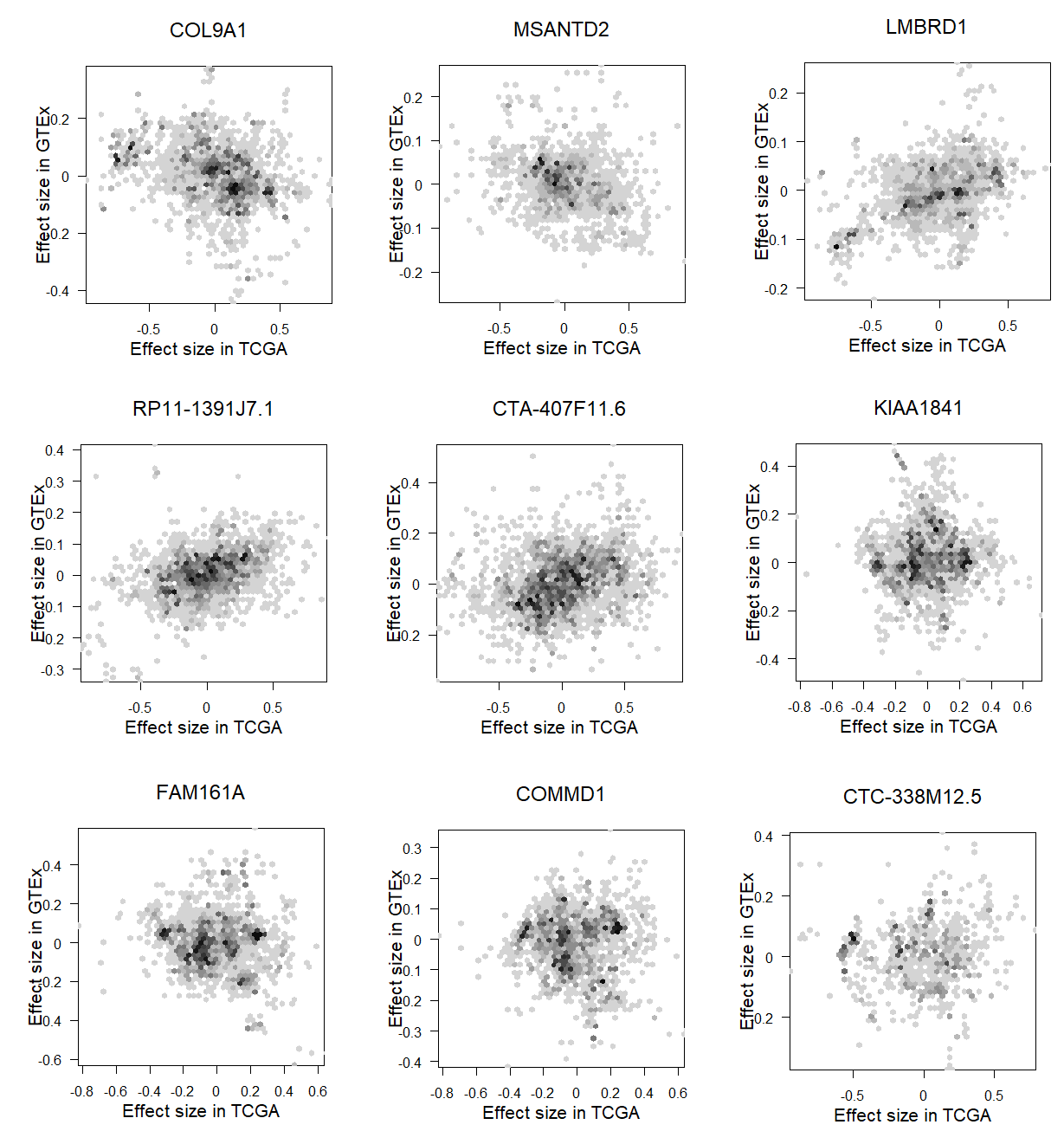


Figure S12. Scatter plots of marginal effect size of SNPs in TCGA and GTEx for gene which were identified by the burden test, the KM test, IEHC-Fisher, IEHC-adapt, IEHC-optim and IEHC-ACAT.


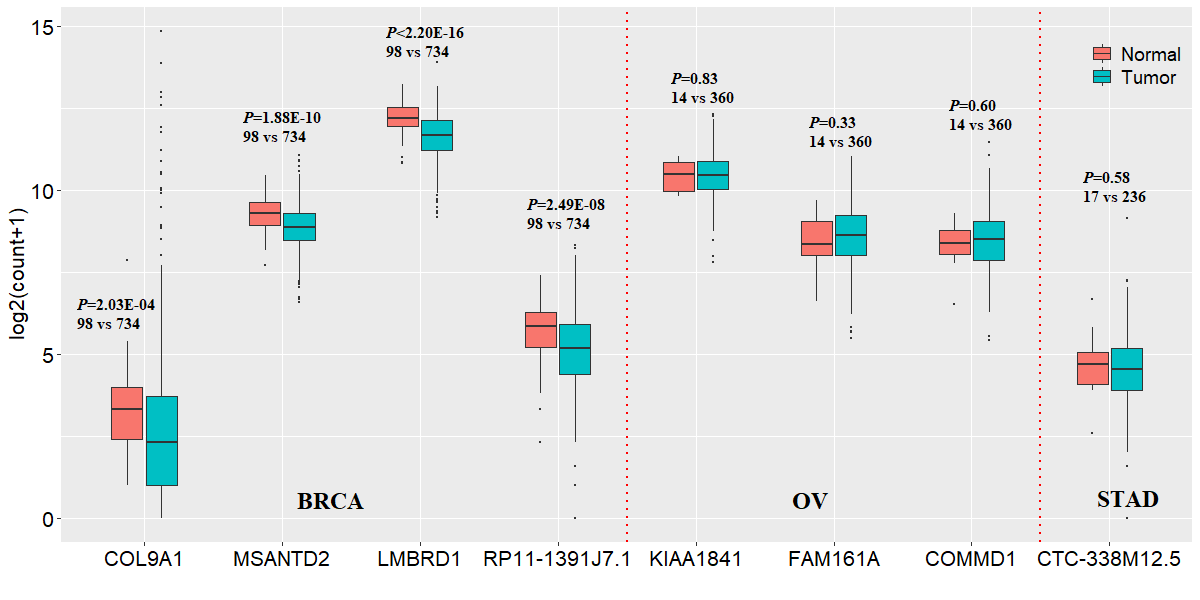


Figure S13. Differential expression levels between tumor samples and patient-matched normal samples for identified genes. The *P* value and the number of tumor samples and patient-matched normal samples are displayed. Because of relatively small sample sizes for normal samples of OV in TCGA, we yielded another ovarian cancer GEO (accession number: GSE18520) dataset which included 53 tumor samples and 10 normal samples from [[19](#_ENREF_19)]. Gene expressions of OV in TCGA and GSE18520 were aligned according to gene symbols; and ComBat [[20](#_ENREF_20)] was used to adjust batch effects between the two datasets using R package sva [[21](#_ENREF_21)]. Of note, the expression level of *CTA-407F11.6* cannot be available in TCGA for COAD; thus, this gene is not considered here.


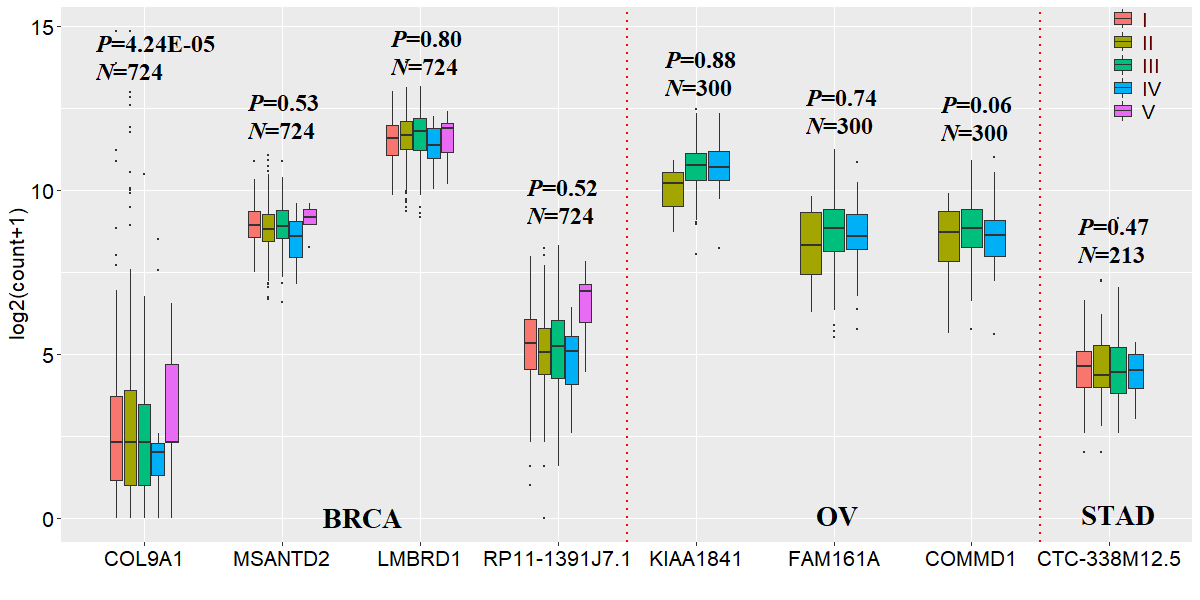


Figure S14. Differential expression levels across various tumor stages for identified genes. When the pathologic tumor stage was unavailable, we alternatively employed the clinical stage (i.e., OV). The *P* value and the number of tumor samples are shown. Of note, the expression level of CTA-407F11.6 is not available in TCGA for COAD; thus, this gene is not considered here*.*

Table S1. The number of genes determined by whether the FDR of regression coefficients and the p-values of IEHC-Fisher are less than 0.05

| Cancer | FDR < = 0.05  IEHC-Fisher < = 0.05 | FDR < = 0.05  IEHC-Fisher > 0.05 | FDR > 0.05  IEHC-Fisher < = 0.05 | FDR > 0.05  IEHC-Fisher > 0.05 | p-value |
| --- | --- | --- | --- | --- | --- |
| ACC | 259 | 8,274 | 19 | 2,611 | 4.61×10^-11^ |
| BRCA | 280 | 4,985 | 54 | 2,464 | 1.52×10^-10^ |
| COAD | 341 | 8,163 | 48 | 2,983 | 3.08×10^-10^ |
| LIHC | 250 | 6,146 | 39 | 2,137 | 3.22×10^-6^ |
| LUAD | 344 | 7,573 | 36 | 2,663 | 5.51×10^-13^ |
| LUSC | 256 | 6,748 | 32 | 2,401 | 1.12×10^-8^ |
| OV | 405 | 4,924 | 143 | 2,247 | 1.21×10^-2^ |
| PAAD | 238 | 7,667 | 31 | 2,790 | 3.69×10^-8^ |
| STAD | 300 | 6,196 | 71 | 2,679 | 6.70×10^-6^ |
| UCEC | 411 | 7,675 | 62 | 2,798 | 6.32×10^-11^ |

Table S2. The number of genes determined by whether the FDR of regression coefficients and the p*-*values of IEHC-adapt are less than 0.05

| Cancer | FDR < = 0.05  IEHC-adapt < = 0.05 | FDR < = 0.05  IEHC-adapt > 0.05 | FDR > 0.05  IEHC-adapt < = 0.05 | FDR > 0.05  IEHC-adapt > 0.05 | p*-*value |
| --- | --- | --- | --- | --- | --- |
| ACC | 293 | 8,240 | 21 | 2,609 | 1.45×10^-12^ |
| BRCA | 281 | 4,984 | 48 | 2,470 | 3.01×10^-12^ |
| COAD | 346 | 8,158 | 45 | 2,986 | 2.21×10^-11^ |
| LIHC | 263 | 6,133 | 35 | 2,141 | 5.35×10^-8^ |
| LUAD | 350 | 7,567 | 40 | 2,659 | 3.66×10^-12^ |
| LUSC | 276 | 6,728 | 39 | 2,394 | 4.64×10^-8^ |
| OV | 402 | 4,927 | 146 | 2,244 | 2.63×10^-2^ |
| PAAD | 262 | 7,643 | 29 | 2,792 | 2.16×10^-10^ |
| STAD | 315 | 6,181 | 70 | 2,680 | 5.39×10^-7^ |
| UCEC | 425 | 7,661 | 59 | 2,801 | 1.38×10^-12^ |

Table S3. The number of genes determined by whether the FDR of regression coefficients and the p*-*values of IEHC-optim are less than 0.05

| Cancer | FDR < = 0.05  IEHC-optim < = 0.05 | FDR < = 0.05  IEHC-optim > 0.05 | FDR > 0.05  IEHC-optim < = 0.05 | FDR > 0.05  IEHC-optim > 0.05 | p*-*value |
| --- | --- | --- | --- | --- | --- |
| ACC | 284 | 8,249 | 21 | 2,609 | 5.60×10^-12^ |
| BRCA | 262 | 5,003 | 44 | 2,474 | 1.09×10^-11^ |
| COAD | 325 | 8,179 | 54 | 2,977 | 8.76×10^-8^ |
| LIHC | 248 | 6,148 | 37 | 2,139 | 1.41×10^-6^ |
| LUAD | 332 | 7,585 | 45 | 2,654 | 1.33×10^-9^ |
| LUSC | 264 | 6,740 | 41 | 2,392 | 7.76×10^-7^ |
| OV | 380 | 4,949 | 141 | 2,249 | 5.19×10^-2^ |
| PAAD | 240 | 7,665 | 28 | 2,793 | 3.65×10^-9^ |
| STAD | 302 | 6,194 | 84 | 2,666 | 5.66×10^-4^ |
| UCEC | 392 | 7,694 | 61 | 2,799 | 5.27×10^-10^ |

Table S4. The number of genes determined by whether the FDR of regression coefficients and the p-values of IEHC-ACAT are less than 0.05

| Cancer | FDR < = 0.05  IEHC-ACAT < = 0.05 | FDR < =0.05 IEHC-ACAT > 0.05 | FDR > 0.05  IEHC-ACAT < = 0.05 | FDR > 0.05  IEHC-ACAT > 0.05 | p-value |
| --- | --- | --- | --- | --- | --- |
| ACC | 317 | 8,216 | 21 | 2,609 | 3.84×10^-14^ |
| BRCA | 304 | 4,961 | 62 | 2,456 | 1.56×10^-10^ |
| COAD | 392 | 8,112 | 52 | 2,979 | 1.71×10^-12^ |
| LIHC | 290 | 6,106 | 40 | 2,136 | 2.38×10^-8^ |
| LUAD | 370 | 7,547 | 41 | 2,658 | 3.39×10^-13^ |
| LUSC | 301 | 6,703 | 44 | 2,389 | 2.50×10^-8^ |
| OV | 452 | 4,877 | 155 | 2,235 | 3.01×10^-3^ |
| PAAD | 283 | 7,622 | 34 | 2,787 | 4.47×10^-10^ |
| STAD | 339 | 6,157 | 80 | 2,670 | 1.39×10^-6^ |
| UCEC | 456 | 7,630 | 63 | 2,797 | 1.57×10^-13^ |

### References

1. Cox DR. Regression Models and Life-Tables. Journal of the royal statistical society Series B (Methodological). 1972;34:187-220.

2. Therneau TM, Grambsch PM, Pankratz VS. Penalized survival models and frailty. Journal of computational and graphical statistics. 2003;12:156-175.

3. Sun J, Zheng Y, Hsu L. A unified mixed-effects model for rare-variant association in sequencing studies. Genet Epidemiol. 2013;37:334-344.

4. Lin X, Cai T, Wu MC, Zhou Q, Liu G, Christiani DC, Lin X. Kernel machine SNP-set analysis for censored survival outcomes in genome-wide association studies. Genet Epidemiol. 2011;35:620-631.

5. Cai T, Tonini G, Lin X. Kernel machine approach to testing the significance of multiple genetic markers for risk prediction. Biometrics. 2011;67:975-986.

6. Nicolae DL, Gamazon E, Zhang W, Duan S, Dolan ME, Cox NJ. Trait-associated SNPs are more likely to be eQTLs: annotation to enhance discovery from GWAS. PLoS Genet. 2010;6:e1000888.

7. Su YR, Di C, Bien S, Huang L, Dong X, Abecasis G, Berndt S, Bezieau S, Brenner H, Caan B, et al. A Mixed-Effects Model for Powerful Association Tests in Integrative Functional Genomics. Am J Hum Genet. 2018;102:904-919.

8. GTEx Consortium. The Genotype-Tissue Expression (GTEx) project. Nat Genet. 2013;45:580-585.

9. GTEx Consortium. Genetic effects on gene expression across human tissues. Nature. 2017;550:204-213.

10. Ripatti S, Palmgren J. Estimation of Multivariate Frailty Models Using Penalized Partial Likelihood. Biometrics. 2000;56:1016-1022.

11. Wu MC, Lee S, Cai T, Li Y, Boehnke M, Lin X. Rare-Variant Association Testing for Sequencing Data with the Sequence Kernel Association Test. Am J Hum Genet. 2011;89:82-93.

12. Zeng P, Zhao Y, Liu J, Liu L, Zhang L, Wang T, Huang S, Chen F. Likelihood ratio tests in rare variant detection for continuous phenotypes. Annals of human genetics. 2014;78:320-332.

13. Satterthwaite FE. An approximate distribution of estimates of variance components. Biometrics bulletin. 1946;2:110-114.

14. Davies RB. Algorithm AS 155: The distribution of a linear combination of χ 2 random variables. Journal of the Royal Statistical Society Series C (Applied Statistics). 1980;29:323-333.

15. Liu H, Tang Y, Zhang HH. A new chi-square approximation to the distribution of non-negative definite quadratic forms in non-central normal variables. Computational Statistics & Data Analysis. 2009;53:853-856.

16. Lee S, Wu MC, Lin X. Optimal tests for rare variant effects in sequencing association studies. Biostatistics. 2012;13:762-775.

17. Koziol JA, Perlman MD. Combining independent chi-squared tests. Journal of the American Statistical Association. 1978;73:753-763.

18. Fisher RA: Statistical Methods for Research Workers, 5th Edn. Biological monographs and manuals. Edinburgh: Oliver and Boyd Ltd; 1934.

19. Mok SC, Bonome T, Vathipadiekal V, Bell A, Johnson ME, Park D-C, Hao K, Yip DK, Donninger H, Ozbun L. A gene signature predictive for outcome in advanced ovarian cancer identifies a survival factor: microfibril-associated glycoprotein 2. Cancer cell. 2009;16:521-532.

20. Johnson WE, Li C, Rabinovic A. Adjusting batch effects in microarray expression data using empirical Bayes methods. Biostatistics. 2007;8:118-127.

21. Jeffrey TL, W. Evan J, Hilary SP, Elana JF, Andrew EJ, John DS, Yuqing Z, Leonardo CT. sva: Surrogate Variable Analysis. 2019.
